# Increasing the Yield in Targeted Next-Generation Sequencing by Implicating CNV Analysis, Non-Coding Exons and the Overall Variant Load: The Example of Retinal Dystrophies

**DOI:** 10.1371/journal.pone.0078496

**Published:** 2013-11-12

**Authors:** Tobias Eisenberger, Christine Neuhaus, Arif O. Khan, Christian Decker, Markus N. Preising, Christoph Friedburg, Anika Bieg, Martin Gliem, Peter Charbel Issa, Frank G. Holz, Shahid M. Baig, Yorck Hellenbroich, Alberto Galvez, Konrad Platzer, Bernd Wollnik, Nadja Laddach, Saeed Reza Ghaffari, Maryam Rafati, Elke Botzenhart, Sigrid Tinschert, Doris Börger, Axel Bohring, Julia Schreml, Stefani Körtge-Jung, Chayim Schell-Apacik, Khadijah Bakur, Jumana Y. Al-Aama, Teresa Neuhann, Peter Herkenrath, Gudrun Nürnberg, Peter Nürnberg, John S. Davis, Andreas Gal, Carsten Bergmann, Birgit Lorenz, Hanno J. Bolz

**Affiliations:** 1 Bioscientia Center for Human Genetics, Ingelheim, Germany; 2 Division of Pediatric Ophthalmology, King Khaled Eye Specialist Hospital, Riyadh, Saudi Arabia; 3 Department of Ophthalmology, Justus-Liebig-University Giessen, University Hospital Giessen and Marburg GmbH, Giessen Campus, Giessen, Germany; 4 Department of Ophthalmology, University of Bonn, Bonn, Germany; 5 Human Molecular Genetics Laboratory, Health Biotechnology Division, National Institute for Biotechnology and Genetic Engineering, Faisalabad, Pakistan; 6 Institute of Human Genetics, University of Lübeck, Lübeck, Germany; 7 Institute of Human Genetics, University Hospital of Cologne, Cologne, Germany; 8 Center for Molecular Medicine Cologne (CMMC), University of Cologne, Cologne, Germany; 9 Cologne Excellence Cluster on Cellular Stress Responses in Aging-Associated Diseases (CECAD), University of Cologne, Cologne, Germany; 10 MRC Holland, Amsterdam, The Netherlands; 11 Comprehensive Genetic Center, Tehran University of Medical Sciences, Tehran, Iran; 12 Avicenna Biotechnology Research Institute, Tehran, Iran; 13 Pränatalzentrum Hamburg und Humangenetik, Hamburg, Germany; 14 Institute of Clinical Genetics, Technical University Dresden, Dresden, Germany; 15 Division of Human Genetics, Medical University Innsbruck, Innsbruck, Austria; 16 Humangenetik, Bremen, Germany; 17 Institute of Human Genetics, Westfälische Wilhelms-University, Münster, Germany; 18 Praenatal-Medizin und Genetik Düsseldorf, Düsseldorf, Germany; 19 Praxis für Humangenetik am DRK-Klinikum Westend, Berlin, Germany; 20 Princess Al Jawhara Albrahim Center of Excellence in Research of Hereditary Disorders, King Abdulaziz University, Jeddah, Saudi Arabia; 21 Medizinisch Genetisches Zentrum, Munich, Germany; 22 Department of Pediatrics, University Hospital of Cologne, Cologne, Germany; 23 Cologne Center for Genomics and Center for Molecular Medicine, University of Cologne, Cologne, Germany; 24 Department of Ophthalmology, Zayed Military Hospital, Abu Dhabi, United Arab Emirates; 25 Institute of Human Genetics, University Medical Center Hamburg-Eppendorf, Hamburg, Germany; 26 Center for Clinical Research, University Hospital of Freiburg, Freiburg, Germany; National Eye Institute, United States of America

## Abstract

Retinitis pigmentosa (RP) and Leber congenital amaurosis (LCA) are major causes of blindness. They result from mutations in many genes which has long hampered comprehensive genetic analysis. Recently, targeted next-generation sequencing (NGS) has proven useful to overcome this limitation. To uncover “hidden mutations” such as copy number variations (CNVs) and mutations in non-coding regions, we extended the use of NGS data by quantitative readout for the exons of 55 RP and LCA genes in 126 patients, and by including non-coding 5′ exons. We detected several causative CNVs which were key to the diagnosis in hitherto unsolved constellations, e.g. hemizygous point mutations in consanguineous families, and CNVs complemented apparently monoallelic recessive alleles. Mutations of non-coding exon 1 of *EYS* revealed its contribution to disease. In view of the high carrier frequency for retinal disease gene mutations in the general population, we considered the overall variant load in each patient to assess if a mutation was causative or reflected accidental carriership in patients with mutations in several genes or with single recessive alleles. For example, truncating mutations in *RP1*, a gene implicated in both recessive and dominant RP, were causative in biallelic constellations, unrelated to disease when heterozygous on a biallelic mutation background of another gene, or even non-pathogenic if close to the C-terminus. Patients with mutations in several loci were common, but without evidence for di- or oligogenic inheritance. Although the number of targeted genes was low compared to previous studies, the mutation detection rate was highest (70%) which likely results from completeness and depth of coverage, and quantitative data analysis. CNV analysis should routinely be applied in targeted NGS, and mutations in non-coding exons give reason to systematically include 5′-UTRs in disease gene or exome panels. Consideration of all variants is indispensable because even truncating mutations may be misleading.

## Introduction

Retinal dystrophies result from degeneration of photoreceptor and retinal pigment epithelium cells. With a prevalence of ∼1 in 3,000, they represent the major cause of hereditary blindness in developed countries [1]. Apart from the individual burden, retinal dystrophies significantly contribute to healthcare costs [2]. Retinal dystrophies are characterized by extensive genetic heterogeneity, with more than 60 genes currently known to underlie retinitis pigmentosa (RP), the most prevalent subtype that affects more than 1.5 million people worldwide [3], [4]. Knowing the causative mutation is desirable for several reasons: It provides the basis for personalized genetic counseling and specification of the recurrence risk, and it may predict the natural clinical course (including the determination of a genetic syndrome). In clinically atypical presentations or ambiguous family history, the genotype may specify or even reverse the previous diagnosis or the assumed mode of inheritance. Regarding the progress of gene-replacement therapy approaches for several retinal dystrophies, the genetic diagnosis will be an essential prerequisite for gene-specific therapies [3], [5]. However, apart from the c.2991+1655A>G mutation in *CEP290* previously reported to be present in 20% of patients with Leber congenital amaurosis (LCA) and *RPGR* in male RP patients [6], [7], there is no major mutation or disease gene for RP and LCA, and clear-cut genotype-phenotype correlations are largely lacking, which prevents efficient targeted Sanger sequencing. Because chip-based analysis for previously reported mutations detects only a fraction of the causative alleles [8], and gene-by-gene analysis by Sanger sequencing is too laborious and expensive, genetic testing has been the exception until recently. Now, next-generation sequencing (NGS) allows for simultaneous and efficient analysis of all known disease genes for a given trait.

NGS of 55 genes involved in RP and LCA (the term “LCA” was applied for early-onset retinal dystrophies, including infant RP and infant cone-rod dystrophies, CRD; Additional Data File S1) in 126 patients. Causative mutations, including CNVs affecting one to multiple exons, were identified in the majority of patients and confirmed the extensive genetic heterogeneity. Our findings demonstrate the immense potential of NGS for diagnostics of retinal dystrophies and shed light on the genetic complexity of this disease group.

## Results and Discussion

### Performance of Two NGS Platforms in RD Gene Panel Analysis

Initially 79 samples were sequenced on the Roche 454 GS FLX platform, followed by 38 samples sequenced on the Illumina MiSeq system. With the Roche platform, 90% of the target exons were covered more than 15-fold, with an average coverage of 75-fold per sample. With the Illumina MiSeq instrument, the average coverage was significantly higher (250-fold) and more complete (15-fold for more than 99% of target sequences). 37% of the samples sequenced on the 454 platform were mutation-negative (29 of 79 samples), compared to only 18% sequenced on the MiSeq (7 of 38 samples). CNV analysis was only possible with high-coverage NGS as obtained with the MiSeq system.

### High Mutation Detection Rate, Extensive Genetic Heterogeneity and Predominance of Novel Mutations

**Figure 1:**
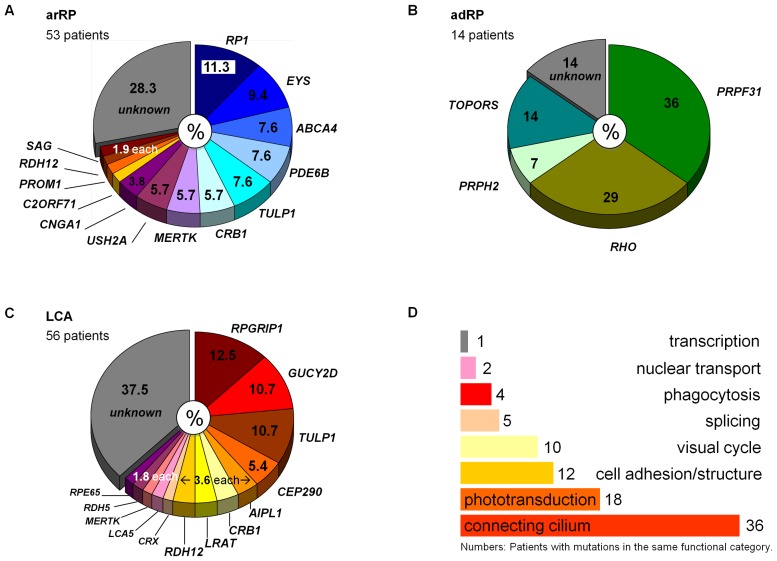
Mutational spectrum in RP and LCA patients. Percentages refer to patients with mutations in the respective gene that are considered causative. The distribution of causative mutations across many genes, each contributing a relatively small fraction to the mutational spectrum, confirms the extensive genetic heterogeneity of retinal dystrophies. Note that the three patients that were found to carry X-linked mutations are not contained in the schemes A – B. **A.** arRP. **B.** adRP. Note that the percentages refer to a relatively small adRP cohort in this study. **C.** LCA. **D.** Functional categorization of genes that were found to carry causative mutations in our study. Mutations in genes encoding components of the photoreceptor’s connecting cilium and associated structures were predominant.

The overall mutation detection rate was 70% (88/126 patients). More specifically, causative mutations were detected in 38/53 patients (72%) with autosomal recessive (ar) and in 12/14 (86%) with autosomal dominant (ad) RP (Figure 1A,B). Three patients turned out to have X-linked RP based on the genetic findings. In LCA, causative mutations were identified in 35/56 patients (63%; Figure 1C). Although mutations in some genes (*RP1* and *EYS* in arRP, and *RPGRIP1*, *GUCY2D* and *TULP1* in LCA) were more prevalent, mutations in many rare genes account for the majority of patients, confirming that these phenotypes are genetically highly heterogeneous and only comprehensively accessible by highly parallel sequencing of all known disease genes. *CEP290*, previously reported as the predominant LCA gene, was not a major contributor to this phenotype in our cohort, and its hot spot mutation, c.2991+1655A>G, was not found at all. This may partially be due to the ethnic background of LCA patients in our cohort with 43% of patients originating from the Arabian peninsula. In contrast to other large studies [9], *USH2A* mutations contributed only to a small proportion of arRP. Causative mutations were found in 28 different genes that encode proteins from diverse pathways and cellular compartments. Mutations in ciliary genes were most prevalent (Figure 1D), indicating the importance of the photoreceptor’s connecting cilium, its associated structures and functions (such as intraflagellar transport) for visual integrity. Of 98 different mutations, 67 were novel (68%) and would thus have been missed by approaches exclusively targeting known alleles such as genotyping microarrays. Below, we describe several families with peculiar findings that further expand our understanding of RD genetics beyond the mere identification of the causative mutations.

### CNV Detection from High-coverage NGS Data

Virtually any gene may be captured and subjected to NGS aimed not only at qualitative, but also quantitative readout. This utilization of NGS data enables CNV detection and can favourably complement MLPA (multiplex ligation-dependent probe amplification), where the application depends on the availability of commercial kits that currently cover only a fraction of known RD genes. We identified four alleles with pathogenic CNVs comprising one to multiple exons. Below, we describe exemplary constellations with CNVs contributing to retinal disease.

#### CNV and point mutation in a non-coding *EYS* exon contributing to arRP

**Figure 2:**
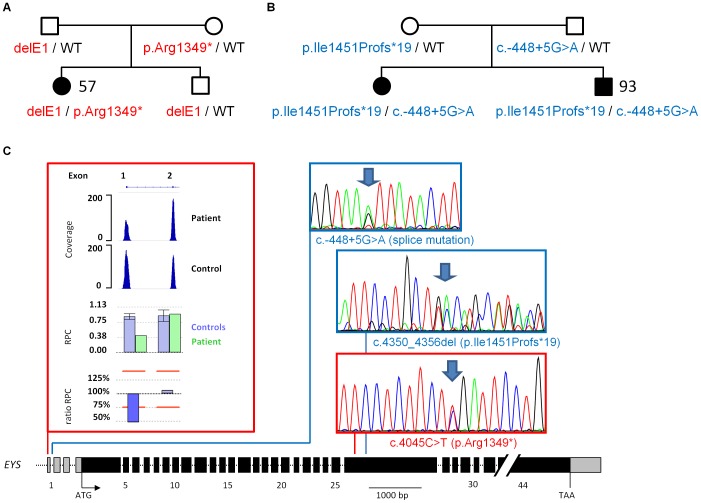
Evidence for mutations in non-coding exon 1 of *EYS* contributing to arRP. Compound-heterozygosity for truncating mutations in the coding sequence of *EYS* and mutations of exon 1 co-segregate with arRP in two families. **A.** Patient 57 carries a deletion of exon 1 *in trans* to a nonsense mutation, whereas patient 93 and his sister **B.** carry a mutation of the donor splice site and a truncating frameshift mutation. **C.** Scheme of the *EYS* gene with non-coding (light grey) and coding (black) exons. Mutations of patients 57 (red) and 93 (blue) are indicated. The heterozygous deletion of exon 1 in patient 57 was detected by quantitative analysis of NGS data. The coverage plot illustrates the statistical readout, with the absolute coverage deduced from unique read count (scale bar, upper panel) and as calculated by the CNV analysis mode in SeqNext (JSI Medical Systems, lower panel). Normalized relative coverage (relative product coverage, RPC) of every target region of interest (ROI) of patient sample (green, RPC P.) and average relative target coverage of control samples (blue, RPC C.). Error bars: standard deviation of control samples. Ratio RPC was calculated from patient’s versus controls’ RPC; ratios below 75% indicate a heterozygous deletion. Electropherograms show the confirmation of the three point mutations by Sanger sequencing (arrows: position of the mutations).

Mutations in *EYS* account for 5–18% of arRP cases depending on the population [10], [11]. It has been suggested that at least 15% of patients with monoallelic point mutations may carry midsized rearrangements as second mutant alleles [12]. In our study, *EYS* mutations were found in 9.4% of arRP patients (five families). One patient was compound heterozygous for a truncating mutation in the coding region and a deletion of non-coding exon 1 at least (Figure 2A,C; Figure S2A in File S1). 5′ non-coding gene sequences, especially first exons, usually contain the promoter and are thus important for gene regulation and vulnerable to mutations [13]. In a recent example, a recurrent *de novo* mutation creating an aberrant initiation codon of the *IFITM5* gene was found to cause a genetic subtype of osteogenesis imperfecta [14], [15]. Promoter site prediction programs TSSG [16] and NNPP [17] predict the *EYS* transcription start site at the beginning of exon 1 and the TATA box upstream. The potential disease-causing effect of exon 1 mutations in *EYS* is supported by two siblings of a second family with a putative splice site mutation of exon 1 *in trans* to a truncating mutation in a coding exon (Figure 2B,C). We therefore propose that loss and aberrant splicing of *EYS* exon 1 should impair transcription of the mutant gene copy and result in a null allele. Our findings illustrate the potential benefit of including 5′-UTRs in NGS of disease gene or even exome panels. Evaluation of non-coding regulatory regions may identify the “missing hit” in heterozygous carriers of recessive mutations.

#### Hemizygosity of a *CRX* mutation in a consanguineous LCA family

**Figure 3:**
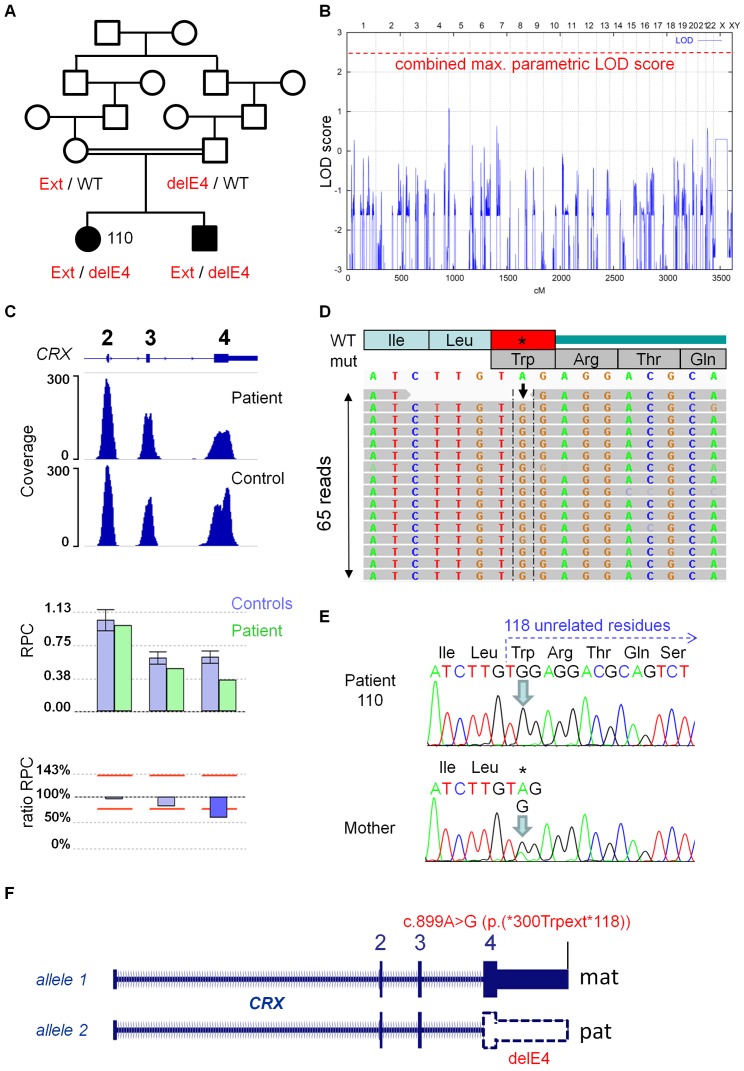
Hemizygosity of a *CRX* mutation in a recessive consanguineous LCA family. **A.** Compound-heterozygosity for a potentially protein-extending no-stop mutation (c.899A>G/p.(*300Trpext*118); here designated as Ext) abrogating the natural termination codon in exon 4 and a deletion of the same exon (delE4) *in trans* in patient 110 and her brother. **B.** Graphical view of the LOD score calculation from genomewide SNP mapping for this family previous to NGS testing: Genomewide homozygosity mapping prior to NGS did not identify a clear candidate locus. The combined maximum parametric LOD score of 2.4 was not obtained. **C.** Scheme of the *CRX* gene and coverage plots for CNV analysis from NGS data (Illumina MiSeq), indicating a heterozygous deletion of exon 4 (upper panel, absolute coverage based on read count; lower panel, SeqNext CNV analysis). See legend to Figure 2C. **D.** Schematic representation of the mapped sequencing reads for the no-stop mutation (Integrative Genomics Viewer). The mutation (arrow) was present in all 65 reads covering this region of the gene and therefore appeared homozygous. **E.** Electropherograms from Sanger sequencing of the no-stop mutation with hemizygosity in patient 110 (upper panel) and heterozygosity in her mother (lower panel). **F.** Summary of the disease-causing genetic constellation in patient 110 and her brother (superimposition on parental alleles).

In a consanguineous Turkish LCA family with two affected siblings (Figure 3A), homozygosity mapping by genome-wide linkage analysis had initially failed to identify an unambiguous chromosomal candidate region, and the combined maximum parametric LOD score of 2.4 was not obtained (Figure 3B). NGS of a sample from the index patient identified an apparently homozygous *CRX* mutation in exon 4 that abrogates the natural translation termination codon (c.899A>G), predicting an elongated protein with 118 unrelated residues (p.*300Trpext*118). Subsequent quantitative analysis revealed a heterozygous deletion of exon 4 *in trans* to the no-stop mutation which was thereby recognized as hemizygous (Figure 3C; Figure S2B in File S1). Both mutations cosegregated with LCA in the family. Interestingly, *CRX* mutations have mostly been observed in autosomal dominant LCA and CRD [18], [19]. Congenital retinal degeneration in a patient with homozygosity for a missense allele, p.Arg90Trp, suggested that *CRX* may also be a recessive LCA gene [20]. The lack of retinal degeneration in both parents of the index patient and LCA in her brother who also carried both mutations strongly indicate that both *CRX* mutations identified here represent recessive loss-of-function alleles, confirming the previous assumption that recessive LCA may result from biallelic *CRX* mutations. This example illustrates how CNV analysis from NGS data can prevent major interpretation pitfalls, especially in consanguineous families with compound heterozygous mutations, including a large deletion simulating the expected homozygosity of a point mutation.

#### CNVs are common in *PRPF31*, an adRP gene to be considered in “simplex” RP

Mutations of *PRPF31* account for about 5–10% of adRP cases (*RP11*) [4], [21]. *RP11* families often display incomplete penetrance, and dominant inheritance may not be obvious from the family history. In five patients, we identified heterozygous *PRPF31* mutations, including deletions of multiple (patient 116) or even all coding exons (patient 113) (see Figure S1 and Figure S2C in File S1). By Sanger sequencing and subsequent MLPA in seven patients with pedigrees suggesting incomplete penetrance, we identified point mutations in two patients, and three had multiple exon to whole-gene deletions (these patients were not part of this study), compatible with a previous study suggesting that the *RP11* locus is prone to genomic rearrangements [22]. Patients 22, 23 and 116 had a provisional diagnosis of sporadic and thus recessive RP which was revised after the genetic findings – resulting in significantly higher recurrence risks of up to 50% for the patients’ offspring to be communicated in genetic counseling. Evaluation of *PRPF31*, including CNV analysis, is therefore advisable in all RP patients independent of the assumed inheritance mode.

### Oligogenic Heterozygosity: Accidental Carriership, Potential Modifiers and Non-pathogenic Truncating Mutations

Given the multitude of genes implicated in RP and LCA, it is not surprising that NGS, providing a “full picture” of the mutational load, identifies constellations with mutations in several genes. In view of a recent study of genome sequences from 46 control individuals from various regions of the world indicating that one in 4–5 individuals from the general population may be a carrier of null mutations in a gene for inherited retinal degeneration [23], constellations with mutations in multiple loci need to be anticipated in a comprehensive NGS approach. In our study, many patients with causative biallelic mutations carried singular heterozygous missense variants in other RD genes (Table 1). These additional alleles were frequently indicated as likely protein-damaging by the prediction programs applied herein, and their contribution to disease severity as modifiers or in an oligo−/digenic setting cannot be excluded. Digenic inheritance has been reported for non-syndromic RP due to double heterozygosity for recessive mutations in *RDS* and *ROM1*, both encoding interacting structural components of rod outer segments [24], and for deafblindness with mutations in genes encoding interacting proteins (*GPR98* and *PDZD7*) of the Usher protein interactome [25]. However, a final proof of causative oligogenic constellations is often impossible because it usually requires segregation analysis and precise phenotyping in extended families, determination of the variants’ prevalence in large cohorts or simulation in animal models as previously reported for *AHI1* and *PDZD7* [25], [26]. Although oligogenic inheritance cannot be excluded in some families, there was no clear evidence for digenic disease or a modifying effect in any patient from our cohort.

**Table 1:** Causative mutations and putatively pathogenic variants identified in this study.

Patient	Gene	Allele 1				Allele 2				Additional Allele				Gender	Age (years)	Phenotype	Inheritance (family history)	Consang.	Origin
		Cds	Protein	db SNP	Ref	cds	Protein	dbSNP	Ref	Cds	Protein	dbSNP	Ref						
Autosomal recessive retinitis pigmentosa, arRP																			
2	*ABCA4*	c.768G>T	p.V256V	–	[1], [2], [3]	c.5603A>T	p.Asn1868Ile	rs1801466	[4], [5]					m	60	RP	ar/s	no	Cau
			(splice)																
30	*ABCA4*	c.1622T>C/	p.Leu541Pro/	rs61751392		c.3210_3211	p.Ser1071Cys			*ABCA4:*				f	17	RP	ar/s	no	Ger
		c.3113C>T	p.Ala1038Val	rs61751374	[6], [7], [8]	dupGT	fs*14	–	[9]	c.5603A>T	p.Asn1868Ile	rs1801466	[4], [5]						
31	*ABCA4*	c.768G>T	p.V256V	–	[1], [2], [3]	c.1622T>C/	p.Leu541Pro/	rs61751392	[6], [7], [8]					f	8	RP	ar/s	no	Ger
			(splice)			c.3113C>T	p.Ala1038Val	rs61751374											
53	*ABCA4*	c.1A>G	p.Met1Val	–	[10]	c.1A>G	p.Met1Val	–	[10]	*ABCA4:*				f	44	RP	ar/s	yes	Ger
			(p.Met1?)				(p.Met1?)			c.6089G>A	p.Arg2030Gln	rs61750641	[10], [11]						
										c.6089G>A	p.Arg2030Gln	rs61750641	[10], [11]						
3	*C2ORF71*	c.2756_2768del	p.Lys919Thr	–	[12]	c.2756_2768del	p.Lys919Thrf	–	[12]					f	44	RP	ar/s	yes	Tur
			fs*2				s*2												
45	*CNGA1*	c.1036C>T	p.Arg346Trp	–	[a]	c.1166C>T	p.Ser389Phe	rs62625014	[13]	*PDE6B:*				m	43	RP	ar/s	no	Ger
										c.703C>T	p.Arg235Cys	–	[a]						
74	*CNGA1*	c.2195A>G	p.Glu732Gly	–	[a]	c.2195A>G	p.Glu732Gly	–	[a]	*TTC8*:				m	50	RP	ar/s	no	Ger
										c.1253A>G	p.Gln418Arg	rs142938748	[a]						
5	*CRB1*	c.1459T>C	p.Ser487Pro	–	[a]	c.1459T>C	p.Ser487Pro	–	[a]	*SAG:*				m	n.d.	RP	ar/s	yes	Pak
										c.374C>T	p.Thr125Met	rs137886124	[a]						
7	*CRB1*	c.2042G>A	p.Cys681Tyr	rs62636266	[14]	c.2308G>C	p.Gly770Arg	–	[a]	*FSCN2:*				f	28	RP	ar/s	no	Pol
										c.805C>T	p.Arg269Cys	–	[a]						
48	*CRB1*	c.2367T>A	p.Asn789Lys	–	[a]	c.2401A>T	p.Lys801*	–	[15]					f	37	RP	ar/s	no	Ger
8	*EYS*	c.604T>C	p.Cys202Arg	–	[a]	c.4350_4356del	p.Ile1451Pro	–	[16]	*MERTK:*				m	52	RP	ar/s	no	Au
							fs*3			c.791C>G	p.Ala264Gly	–	[a]						
										*SAG:* c.473C>A	p.Thr158Lys	–	[a]						
34	*EYS*	c.7055+1G>T	splice	–	[a]	c.7055+1G>T	splice	–	[a]					f	40	RP	ar/s	yes	Syr
57	*EYS*	c.4045C>T	p.Arg1349*	–	[a]	deletion of	?	–	[a]					f	32	RP	ar/s	no	Ger
						exon 1													
88	*EYS*	c.67delA	p.Thr23His	–	[a]	c.162C>A	p.Tyr54*	–	[a]					m	48	RP	ar/Xl	no	Ger
			fs*19																
93	*EYS*	c.4350_4356del	p.Ile1451Pro	–	[16]	c.−448+5G>A	splice	–	[a]	*RD3:*				m	23	RP	ar/s	no	Ger
			fs*3							c.584A>T	p.Asp195Val	rs143207434	[a]						
9	*MERTK*	c.345C>G	p.Cys115Trp	–	[a]	c.1530delT	p.Cys510Trp	–	[a]					m	19	RP	ar/Xl	no	Ger
							fs*5												
46	*MERTK*	c.1786G>A	p.Gly596Arg	–	[a]	c.1786G>A	p.Gly596Arg	–	[a]					f	36	RP	ar/s	n.d.	n.d.
76	*MERTK*	c.1450G>A	p.Gly484Ser	–	[a]	c.1450G>A	p.Gly484Ser	–	[a]	*RP2:*				m	30	RP	ar/s	no	Italy
										c.844C>T	p.Arg282Trp	rs1805147	[17], [18]						
										*RHO:*									
										c.310G>A	p.Val104Ile	rs144317206	19]						
20	*PDE6B*	c.669T>A	p.Tyr223*	–	[a]	c.669T>A	p.Tyr223*	–	[a]	*RBP3:*				f	30	RP	ar/s	yes	Iran
										c.2092C>A	p.Arg698Ser	–	[a]						
21	*PDE6B*	c.1699C>T	p.Gln567*	–	[a]	c.1699C>T	p.Gln567*	–	[a]	*ABCA4:*				f	36	RP	ar/s	yes	Ger
										c.5603A>T	p.Asn1868Ile	rs1801466	[4], [5]						
62	*PDE6B*	c.2193+1G>A	splice	–	[20]	c.2193+1G>A	splice	–	[20]	*ABCA4*:				m	47	RP	ar/s	yes	Ger
										c.2184C>A	p.Ser728Arg	–	[a]						
75	*PDE6B*	c.2193+1G>A	splice	–	[20]	c.2047G>A	p.Val683Met	–	[a]	*PDE6B:*				m	45	RP	ar/s	no	Ger
										c.2249T>G	p.Val750Gly	–	[a]						
55	*PROM1*	c.642T>A	p.Tyr214*	–	[a]	c.1209_1229del	p.Gln403_Ser	–	[a]	*RP1:*				m	26	RP	ar/s	no	Ger
							410delinsHis			c.6304C>T	p.Gln2102*	–	[a]						
										*CRB1:*									
										c.2042G>A	p.Cys681Tyr	rs62636266	[14]						
										*RPGRIP1:*									
										c.1767G>T	p.Gln589His	rs34067949	[21], [22]						
89	*RDH12*	c.226G>C	p.Gly76Arg	–	[23]	c.869T>G	p.Val290Gly	rs61740289	[a]	*ABCA4:*				f	34	RP	ar/s	yes	DRC
										c.618C>G	p.Ser206Arg	rs61748536	[24]						
										*C2ORF71*:									
										c.1844T>A	p.Val615Asp	rs140776870	[25]						
25	*RP1*	c.597C>A	p.Tyr199*	–	[a]	c.3157delT	p.Tyr1053Thr	–	[26]	*CDH23:*				f	38	RP	ar/s	no	Ger
							fs*4			c.6322G>T	p.Glu2108*	–	[27]						
										*CRB1:*									
										c.3122T>C	p.Met1041Thr	rs62635656	[28]						
26	*RP1*	c.4242_4243del	p.His1414Gln	–	[a]	c.4474G>T	p.Glu1492*	–	[a]					f	47	RP	ar/s	yes	Iran
			fs*5																
27	*RP1*	c.1012C>T	p.Arg338*	–	[a]	c.1012C>T	p.Arg338*	–	[a]	*ABCA4:*				f	37	RP	ar/s	yes	Iran
										c.5603A>T	p.Asn1868Ile	rs1801466	[4], [5]						
28	*RP1*	c.5278_5287del	p.Asn1760Cys	–	[a]	c.5278_5287del	p.Asn1760Cys	–	[a]					f	12	RP	ar/s	yes	Tur
			fs*46				fs*46												
36	*RP1*	c.607G>A	p.Gly203Arg	–	[a]	c.607G>A	p.Gly203Arg	–	[a]					m	n.d.	RP	ar/s	yes	Iran
101	*RP1*	c.3843delT	p.Pro1282Leu	–	[a]	c.3843delT	p.Pro1282Leu	–	[a]					f	37	RP	ar/s	yes	Tur
			fs*12				fs*12												
29	*SAG*	c.577C>T	p.Arg193*	–	[29]	?								m	16	RP	ar/s	no	Ger
33	*TULP1*	c.371_394del	p.Asp124_	–	[a]	?				*CRX:*				m	52	RP	ar/s	no	Ger
			132delinsAla							c.122G>A	p.Arg41Gln	rs61748436	[30]						
										*CRX:*									
										c.425A>G	p.Tyr142Cys	rs61748442	[21]						
82	*TULP1*	c.371_394del	p.Asp124_	–	[a]	?								f	35	RP	ar/s	no	Ger
			132delinsAla																
37	*TULP1*	c.1047T>G	p.Asn349Lys	–	[a]	c.1047T>G	p.Asn349Lys	–	[a]	*PDE6A:*				f	n.d.	RP	ar/s	yes	Iran
										c.923C>T	p.Pro308Leu	–	[a]						
38	*TULP1*	c.1198C>T	pArg400Trp	–	[31]	c.1198C>T	pArg400Trp	–	[31]	*ABCA4:*				f	n.d.	RP	ar/s	yes	Iran
										c.2353C>T	p.Arg785Cys	–	[a]						
										*FSCN2:*									
										c.325C>T	p.Arg109Cys	–	[a]						
13	*USH2A*	c.10421A>G	p.Tyr3474Cys	–	[a]	c.13257_13263	p.Phe4419Leu	–	[a]					m	26	RP	ar/s	no	Ger
						del	fs*2												
18	*USH2A*	c.6925T>C	p.Cys2309Arg	–	[a]	c.10561T>C	p.Trp3521Arg	rs111033264	[32], [33]	*ABCA4:*				f	50	RP	ar/s	no	Ger
										c.5603A>T	p.Asn1868Ile	rs1801466	[4], [5]						
										*AIPL1:*									
										c.401A>T	p.Tyr134Phe	rs16955851	[34]						
68	*USH2A*	c.1256G>T	p.Cys419Phe	rs121912600	[35]	c.10342G>A	p.Glu3448Lys	–	[a]					m	44	RP	ar/s	no	Ger
**Autosomal dominant RP, adRP**																			
22	*PRPF31*	c.1048C>T	p.Gln350*	–	[a]					*RPE65:*				f	38	RP	ar/s	no	E-Eur
										c.963T>G	p.Asn321Lys	rs149916178	[36]						
23	*PRPF31*	c.1067_1073+8	Splice	–	[a]									f	36	RP	ar/s	no	Ger
		del																	
43	*PRPF31*	c.217A>T	p.Lys73*	–	[a]									m	18	RP	ad	no	Ger
113	*PRPF31*	Deletion of	haplo-	–	[37], [38]					*ABCA4:*				f	55	RP	ad	no	Ger
		exons 1–14	insufficiency							c.1928G>A	p.Val643Gly	rs114572202	[39]						
										c.5603A>T	p.Asn1868Ile	rs1801466	[4], [5]						
										*RPGRIP1:*									
										c.2555G>A	p.Arg852Gln	rs181758389	[21]						
116	*PRPF31*	Deletion of	haplo-	–	[37], [38]									f	14	RP	ar/s	No	Cau
		exons 1–5	insufficiency																
47	*PRPH2*	c.920delT	p.Leu307Arg	–	[40]					*FSCN2*:				m	33	RP	ad	no	Ger
			fs*17							c.412C>T	p.His138Tyr	rs143796236	[a]						
32	*RHO*	c.35C>G	p.Pro12Arg	–	[a]					*RLBP1:*				f	71	RP	ad	no	Ger
										c.545T>G	p.Phe182Cys	rs142244640	[a]						
44	*RHO*	c.541G>A	p.Glu181Lys	–	[41]									f	47	RP	s	no	Ger
92	*RHO*	c.180C>A	p.Tyr60*	–	[a]					*ROM1:*				f	31	RP	ad	no	Ger
										c.178C>A	p.Pro60Thr;	–	[42], [43]						
										c.323C>T	p.Thr108Met	rs146358003	[42], [43]						
										*ABCA4:*									
										c.1654G>A	p.Val552Ile	rs145525174	[44]						
										c.5714+5G>A *RP2:*	splice	rs61751407	[45]						
										c.844C>T									
										*RPGRIP1:* c.2510C>G	p.Arg282Trp	rs1805147	[17], [18]						
																			
											p.Ala837Gly	–	[22]						
121	*RHO*	c.937-1G>T	splice	–	[46], [47]					*ABCA4:*				m	25	RP	ad	no	Cau
										c.5603A>T	p.Asn1868Ile	rs1801466	[4], [5]						
103	*TOPORS*	c.2554_2557del	p.Glu852Gln	–	[a]									f	30	RP	ad	no	Ger
			fs*13																
108	*TOPORS*	c.2550_2553del	p.Asp850Glu	–	[a]					*ABCA4:*				f	59	RP	ad	no	Ger
			fs*15							c.4771G>A	p.Gly1591Arg	rs113106943	[a]						
										*PDE6A:*									
										c.298C>T	p.Arg100Trp	–	[a]						
**X-linked RP**																			
77	*RP2*	c.226G>T	p.Asp76Tyr	–	[a]					*RP2:*				m	15	RP	ar/s	no	SE-Eur
										c.844C>T	p.Arg282Trp	rs1805147	[17], [18]						
										*ABCA4:*									
										c.5882G>A	p.Gly1961Glu	rs1800553	[7], [48]						
100	*RPGR*	c.1853_1856dup	p.Glu621Lys	–	[a]									f	54	RP	ar	no	Ger
			fs*10																
119	*RPGR*	c.1544T>G	p.Leu515*	–	[a]									m	5	LCA	ar	no	KSA
**Leber congenital amaurosis, LCA**																			
1	*AIPL1*	c.834G>A	p.Trp278*	rs62637014	[49]	c.834G>A	p.Trp278*	rs62637014	[49]	*RD3:*				m	2	LCA	ar	possible	Ger
										c.584A>T	p.Asp195Val	rs143207434	[a]						
										*SAG:*									
										c.374C>T	p.Thr125Met	rs137886124	[a]						
83	*AIPL1*	c.50T>C	p.Leu17Pro	–	[50]	c.50T>C	p.Leu17Pro	–	[50]	*CRB1:*				f	10	LCA	ar	possible	Tur
										c.3397G>A	p.Val1133Met	–	[a]						
										*SEMA4A:*									
										c.1301T>C	p.Met434Thr	rs146822426	[a]						
4	*CEP290*	c.3640dupG	p.Glu1214Gly	–	[a]	c.6604delA	p.Ile2202Leu	–	[51]					f	2	LCA	ar	no	Au
			fs*7				fs*24												
80	*CEP290*	c.2578G>T	p.Glu860*	–	[a]	c.3758G>A	p.Arg1253His	–	[a]	*CEP290:*				f	9	LCA (early CRD)	ar	no	Tur
										c.5254C>T	p.Arg1752Trp	–	[a]						
										*FSCN2:*									
										c.377C>T	p.Ser126Phe	–	[a]						
94	*CEP290*	c.6012-2A>G	splice	–	[a]	c.6870delT	p.Gln2291Lys	–	[52]	*ABCA4:*				f	5	LCA	ar	no	KSA
							fs*10			c.1140T>A;	p.Asn380Lys	rs61748549	[4]						
										c.5642C>T;	p.Ala1881Val	–	[53]						
										c.5882G>A	p.Gly1961Glu	rs1800553	[7], [48]						
										*RP1:*									
										c.1380G>C	p.Lys460Asn	rs143494598	[a]						
6	*CRB1*	c.2842+2T>A	splice	–	[a]	c.2842+2T>A	splice	–	[a]					m	20	LCA		no	Tur
63	*CRB1*	c.1180T>C	p.Cys394Arg	–	[a]	c.1180T>C	p.Cys394Arg	–	[a]					m	6	LCA	ar	yes	KSA
110	*CRX*	deletion of exon 4	NMD?	–	[a]	c.899A>G	p.(*300Trp	–	[a]	*RP1:*				f	9	LCA	ar	yes	Tur
							ext*118)			c.3191G>T	p.Ser1064Ile	–	[a]						
										*SPATA7:*									
										c.1112T>C	p.Ile371Thr	rs150364664	[a]						
56	*GUCY2D*	c.1093_1106del	p.Arg365Trp	–	[a]	c.1093_1106del	p.Arg365Trp	–	[a]					m	4	LCA	ar	yes	Tur
			fs*77				fs*77												
71	*GUCY2D*	c.1401dupT	p.Leu468Ser	–	[a]	c.1401dupT	p.Leu468Ser	–	[a]	*RDH12:*				m	5	LCA	ar	n.d.	KSA
			fs*89				fs*89			c.917T>G	p.Val306Gly	–	[a]						
73	*GUCY2D*	c.2766C>G	p.Tyr922*	–	[a]	c.2766C>G	p.Tyr922*	–	[a]	*GUCY2D:*				f	18	LCA	ar	yes	Tur
										c.2927G>T	p.Arg976Leu	rs61750184	[54]						
										c.2927G>T	p.Arg976Leu	rs61750184	[54]						
118	*GUCY2D*	c.2080C>T	p.Gln694*	–	[a]	?								m	5	LCA	ar	no	Ger
123	*GUCY2D*	c.389delC	p.Pro130Leu	rs61749670	[55]	c.389delC	p.Pro130Leu	rs61749670	[55]					f	1	LCA	ar	no	Mor
			fs*36				fs*36												
125	*GUCY2D*	c.1401dupT	p.Leu468Ser	–	[a]	c.1401dupT	p.Leu468Ser	–	[a]	*RDH12:*			[a]	f	5	LCA	ar	yes	KSA
			fs*89				fs*89			c.917T>G	p.Val306Gly	–							
107	*LCA5*	c.763C>T	p.Arg255*	rs151017794	[a]	c.763C>T	p.Arg255*	rs151017794	[a]	*ABCA4*:				f	1	LCA	ar	n.d.	KSA
										c.1927G>A	p.Val643Met	rs61749417	[10]						
										*PDE6B:*									
										c.704G>A	p.Arg235His	–	[a]						
84	*LRAT*	c.233_242del	p.Leu78Arg	–	[a]	c.233_242del	p.Leu78Arg	–	[a]					m	19	LCA	ar	yes	KSA
			fs*85				fs*85												
87	*LRAT*	c.449dupG	p.Phe151Leu	–	[a]	c.449dupG	p.Phe151Leu	–	[a]					f	11	LCA	ar	yes	Tur
			fs*33				fs*33												
70	*MERTK*	c.1744_1751	p.Ile582*	–	[a]	c.1744_1751	p.Ile582*	–	[a]	*RBP3:*				m	16	LCA	ar	yes	Tur
		delinsT				delinsT				c.2789T>C	p.Ile930Thr	–	[a]						
24	*RDH5*	c.602C>T	p.Ser201Phe	–	[a]	c.602C>T	p.Ser201Phe	–	[a]					f	15	LCA (early CRD)	ar	yes	Pak
49	*RDH12*	c.133A>G	p.Thr45Ala	–	[56]	c.133A>G	p.Thr45Ala	–	[56]	*GUCY2D:*				m	12	LCA	ar	no	Ger
										c.2359T>G	p.Cys787Gly	–	[a]						
										*SPATA7:*	p.Met1Ile								
										c.3G>A	(p.Met1?)	–	[57]						
120	*RDH12*	c.188G>T	p.Gly63Val	–	[a]	c.188G>T	p.Gly63Val	–	[a]	*ABCA4:*			[a]	m	7	LCA	ar	yes	KSA
			(splice)				(splice)			c.3482G>T	p.Arg1161Leu	–							
95	*RPE65*	c.271C>T	p.Arg91Trp	rs61752871	[58]	c.271C>T	p.Arg91Trp	rs61752871	[58]	*CDHR1:*				f	28	LCA	ar	n.d.	KSA
										c.1202G>C,	p.Gly401Ala,	–	[a]						
										c.1202G>C	p.Gly401Ala;	–	[a]						
										*LCA5:*									
										c.764G>A	p.Arg255Gln	–	[a]						
35	*RPGRIP1*	c.2608_2609insA	p.Leu870Tyr	–	[a]	c.2608_2609insA	p.Leu870Tyr	–	[a]	*RPE65:*				f	9	LCA	ar	no	Dubai
			fs*7				fs*7			c.394G>A	p.Ala132Thr	rs61752878	[a]						
50	*RPGRIP1*	c.1107delA	p.Glu370Asn	rs61751266	[59]	c.1107delA	p.Glu370Asn	rs61751266	[59]					m	6	LCA	ar	yes	KSA
			fs*5				fs*5												
81	*RPGRIP1*	c.3565C>T	p.Arg1189*	–	[a]	c.3565C>T	p.Arg1189*	–	[a]	*TTC8:*				m	11	LCA	ar	n.d.	KSA
										c.512A>T	p.Asp171Val	–	[a]						
90	*RPGRIP1*	c.1107delA	p.Glu370Asn	rs61751266	[59]	c.1107delA	p.Glu370Asn	rs61751266	[59]	*MERTK:*				m	1	LCA	ar	n.d.	KSA
			fs*5				fs*5			c.2435A>C	p.Tyr812Ser	rs141361084	[a]						
109	*RPGRIP1*	c.2662C>T	p.Arg888*	–	[a]	c.2662C>T	p.Arg888*	–	[a]	*C2ORF71*:				f	6	LCA	ar	yes	KSA
										c.1844T>A	p.Val615Asp	rs140776870	[25]						
111	*RPGRIP1*	c.1107delA	p.Glu370Asn	rs61751266	[59]	c.1107delA	p.Glu370Asn	rs61751266	[59]	*RBP3:*				f	6	LCA	ar	yes	KSA
			fs*5				fs*5			c.490G>A	p.Ala164Thr	–	[a]						
115	*RPGRIP1*	c.1107delA	p.Glu370Asn	rs61751266	[59]	c.1107delA	p.Glu370Asn	rs61751266	[59]	*PROM1:*				f	2	LCA	ar	yes	KSA
			fs*5				fs*5			c.2094C>A	p.Ser698Arg	–	[a]						
96	*TULP1*	c.901C>T	p.Gln301*	–	[60]	c.901C>T	p.Gln301*	–	[60]	*C2ORF71*:				m	7	LCA	ar	yes	KSA
										c.1844T>A	p.Val615Asp	rs140776870	[25]						
112	*TULP1*	c.901C>T	p.Gln301*	–	[60]	c.901C>T	p.Gln301*	–	[60]					m	19	LCA	ar	no	KSA
114	*TULP1*	c.1604T>C	p.Phe535Ser	–	[a]	c.1604T>C	p.Phe535Ser	–	[a]					f	7	LCA	ar	n.d.	UEA
117	*TULP1*	c.901C>T	p.Gln301*	–	[60]	c.901C>T	p.Gln301*	–	[60]					f	8	LCA	ar	yes	KSA
122	*TULP1*	c.901C>T	p.Gln301*	–	[60]	c.901C>T	p.Gln301*	–	[60]					f	3	LCA	ar	yes	KSA
124	*TULP1*	c.901C>T	p.Gln301*	–	[60]	c.901C>T	p.Gln301*	–	[60]	*RP1:*				m	6	LCA	ar	possible	KSA
										c.5248G>T	p.Glu1750*	–	[a]						

Causative alleles are being listed as “allele 1” and “allele 2” in resolved cases. Additional alleles are shown if the minor allele frequency is below 3% and if *in silico* prediction suggests putative pathogenicity. The inheritance pattern was largely delineated from pedigree informations. In patients 22, 23, 77, 100, 116 and 119, the true mode of inheritance had not been evident from the pedigree information and was finally deduced from the genotype. a, this study. References for studies cited in this table can be found in the Supplementary Material (References S1 in File S1). n.d., not defined; f, female; m, male; ar, autosomal recessive; ad, autosomal dominant; s, sporadic. Xl, X-linked. Cau, Caucasian; Ger, Germany; Tur, Turkey; KSA, Kingdom of Saudi Arabia; Pol, Poland; Au, Austria; Syr, Syria; Pak, Pakistan; DRC, Democratic Republic of the Congo; Mor, Morocco; UAE, United Arab Emirates; E-Eur, East Europe; SE-Eur, Southeast Europe.

However, we identified patients with causative biallelic mutations in recessive RP genes who additionally carried heterozygous truncating mutations in secondary loci. *RP1*, the most prevalent arRP gene in our cohort, was frequently found together with mutations in other RD genes. The observed constellations resulted in different deductions regarding the pathogenicity of the respective *RP1* allele:

#### Pathogenic *RP1* truncations with causality in the family

**Figure 4:**
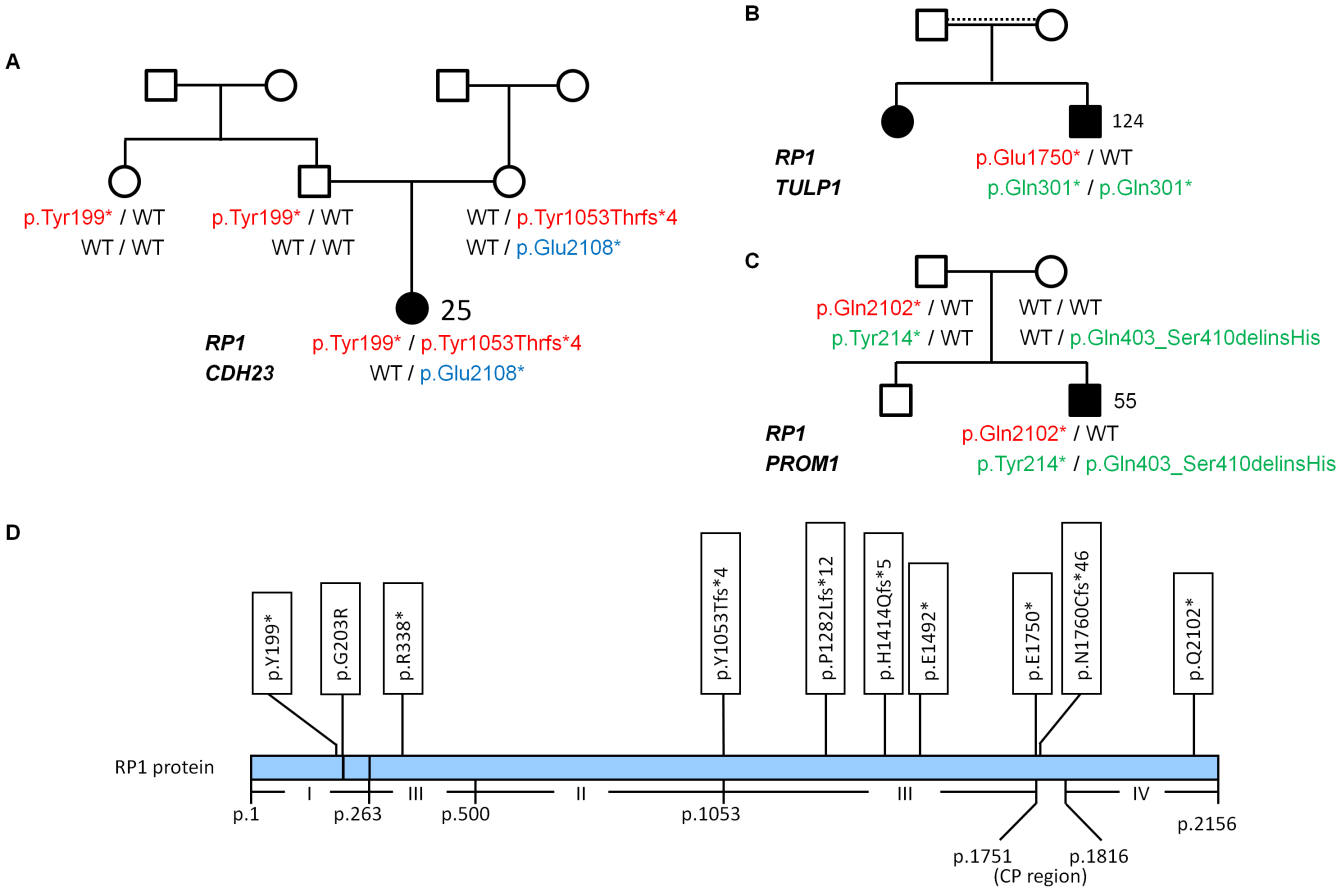
Different arRP scenarios implicating truncating *RP1* mutations with diverse impact on disease. **A.** Pedigree of patient 25 whose arRP is caused by two truncating recessive *RP1* alleles. In addition, the patient carries a heterozygous *CDH23* nonsense mutation that has been reported in USH1 patients but is probably unrelated to disease here. **B.** LCA in patient 124 is due to homozygosity for the founder mutation p.Gln301* in *TULP1*. Heterozygosity for the *RP1* nonsense mutation p.Glu1750* likely reflects accidental carriership. It likely represents a recessive loss-of-function allele. Dotted horizontal line: likely consanguinity. **C.** Compound heterozygosity for two truncating *PROM1* mutations can be considered pathogenic in arRP patient 55. The *RP1* nonsense mutation p.Gln2102* locates near the C-terminus and likely represents an NMD-insensitive non-pathogenic variant. **D.** Scheme of the RP1 protein and overview of truncating *RP1* mutations reported in this study (mutations shown in A – C in red). The four classes of *RP1* truncating mutations [31] are displayed. Class I, NMD-sensitive truncations; class II, NMD-insensitive truncating mutations representing the majority of pathogenic truncation mutations in *RP1* (dominant negative pathomechanism); class III, NMD-insensitive truncation mutations representing loss-of-function arRP mutations; class IV, NMD-insensitive, non-pathogenic truncations located 3′ of p.1816. CP, “critical position”: 65-residue region between p.1751 and p.1816 containing a yet undefined protein residue before which truncation causes disease.

*RP1* mutations are mostly truncating and may cause adRP [27] or arRP [28]. Of note, no *RP1* mutations were observed in our adRP patients, but *RP1* was the most prevalent arRP gene, with clearly causative biallelic mutations in several cases (11,3%; Table 1). Patient 25 was compound-heterozygous for two truncating *RP1* alleles, c.597C>A (p.Tyr199*) and c.3157delT (p.Tyr1053Thrfs*4), and additionally carried a nonsense mutation in *CDH23* which has previously been described in recessive deafblindness (Usher syndrome type 1D, *USH1D*) [29]. Segregation analysis for the mutations in *RP1* and *CDH23*, both encoding proteins of the photoreceptor’s connecting cilium, was compatible with both *RP1* alleles acting recessively (Figure 4A). Detailed ophthalmological investigation revealed no abnormalities in the mother who was double heterozygous for the c.3157delT*_RP1_* mutation and the *CDH23* mutation, excluding a digenic mechanism with an elevated recurrence risk for RP for the patients offspring solely based on her genotype. Although the *CDH23* mutation may modify disease expression in patient 25, her phenotype did not appear unusually severe compared to other patients with *RP1*-associated arRP. Patient 25 can hence be regarded an accidental carrier of the *CDH23* mutation, and her RP is sufficiently explained by her *RP1* mutations. Of note, c.3157delT*_RP1_* has been reported as a dominant mutation in an *RP1* screening study [30]. Based on our data, we assume that adRP in the reported family was possibly due to a mutation in another adRP gene, and the detection of the heterozygous c.3157delT*_RP1_* mutation likely represented supplemental carriership for a recessive allele.

#### Accidental carriers of pathogenic *RP1* truncations and refinement of the *RP1* “critical position”

A monoallelic truncating *RP1* mutation (p.Glu1750*) was found in LCA patient 124, on a homozygous *TULP1* nonsense mutation background (Figure 4B). The *TULP1* mutation was clearly causative. The *RP1* mutation p.Glu1750* flanks a region referred to as “critical position” (Figure 4D) between residues p.1751 and p.1816 that may distinguish between non-functional and functional truncated RP1 proteins [31]: Homozygosity for p.Asn1751Ilefs*3 was shown to cause arRP [32] while homozygosity for the nonsense mutation p.Cys1816* did not evoke a retinal phenotype [33]. Hence, p.Glu1750* very likely presents a pathogenic recessive allele, an interpretation that is compatible with the homozygous mutation p.Asn1760Cysfs*46 segregating with arRP in another consanguineous arRP family from our cohort (patient 28), refining the “critical position” to p.1760– p.1816. However, patient 124 is obviously an accidental carrier of p.Glu1750*_RP1_. The constellation resembles the findings in patient 49 who carries a heterozygous mutation of the *SPATA7* initiation codon in addition to a likely causative homozygous *RDH12* mutation.

#### Non-pathogenic *RP1* truncations

In patient 55, RP was well explained by compound heterozygosity for the *PROM1* mutations p.Tyr214* and p.Gln403_Ser410delinsHis (Figure 4C). In addition, he carried a heterozygous *RP1* nonsense allele, p.Gln2102*, that localized beyond the “critical position” where truncations of C-terminal residues may result in RP1 proteins retaining full function and can be considered non-pathogenic [31], [33].

These three scenarios with *RP1* mutations (a–c) demonstrate that even truncating mutations must be assessed with caution and in the context of the full variant load of known disease genes in order to avoid false interpretations: If a monoallelic truncation is found in addition to clearly causative biallelic mutations in another gene, it may either represent accidental carriership for a pathogenic allele (unrelated to disease in that patient) or a non-pathogenic variant in a non-essential gene region (as is the case at the very C-terminal part of *RP1*).

### Monoallelic Mutations in Genes Underlying Recessive Retinal Dystrophies

Monoallelic mutations in recessive disease genes represent a challenge for interpretation regarding their causality in the patient, especially if there are no biallelic mutations in another gene for the trait that would qualify such mutations as incidental findings (i.e. carrier status unrelated to the disease in the individual). While single non-synonymous variants in recessive disease genes may often represent rare non-pathogenic variants (see Table 1, “Additional Alleles”, and Table S2 in File S1), the nature of the alteration strongly suggests loss of function for two monoallelic mutations in recessive RP genes in our cohort: The same large *in-frame* deletion-insertion mutation in *TULP1* (p.Asp124_132delinsAla) was identified in two independent simplex RP patients (patients 33 and 82), and an *SAG* nonsense mutation was found in patient 29. Patient 33 in addition carried two *CRX* missense variants, p.Arg41Gln and p.Tyr142Cys, that we consider likely benign (both are listed as disease-causing in HGMD, but also in dbSNP, and p.Tyr142Cys was found in several patients with disease-causing mutations in other genes). The sporadic occurrence of RP suggests autosomal recessive inheritance in all three patients, making a dominant-negative effect of the *TULP1* and the *SAG* mutation unlikely. The three DNA samples with monoallelic mutations in *TULP1* and *SAG* mutations were initially sequenced on the GS FLX system; subsequent analysis on the MiSeq platform did not identify additional mutant alleles, in particular no CNVs.

Patients 33, 82 and 29 are therefore either accidental carriers of the *TULP1* and *SAG* mutations with the causative mutation in another arRP gene not known at the time of study design, or the “missing alleles” escaped detection by exonic sequencing because they are deep intronic (as exemplified by the LCA mutation c.2991+1655A>G*_CEP290_* or the only known *RP23* mutation in the *OFD1* gene [34]), or because they localize in regulatory non-coding regions (as shown for *EYS* exon 1 in this study).

### Patients without Mutations – possible Explanations

As discussed above, mutations in known retinal dystrophy genes may escape detection because of their localization – about 15% of disease-causing mutations localize outside coding exonic sequences [35]. Non-coding exons were not systematically included in our study; the identification of mutations in non-coding exon 1 of *EYS* suggests that such exons should be included in upcoming disease gene panels. Mutation-negative cases in our study will in part be due to mutations in RP and LCA genes that were identified after the design of our gene panel (e.g. *NMNAT1*, *DHDDS*, *ZNF513*, *FAM161A*, *KCNJ13*, *IMPG2*, *IQCB1*, *CLRN1*, *MAK*, *C8ORF37*, *PRPF6*, *OFD1*). For example, subsequent exome sequencing for patient 15 identified a homozygous nonsense mutation in *IMPG2* (data not shown). Updating the panel accordingly will identify the causative mutations in additional patients.

Mutations in the X-linked RP genes *RP2* and *RPGR* have been reported to account for 8.5% of cases with RP of apparently autosomal dominant transmission and for 15% of males with simplex retinal degenerative disease [7], [36]. While enrichment and NGS of *RP2* is uncomplicated, the mutational hot spot exon of *RPGR*, *ORF15*, is not accessible by our NGS approach due to its highly repetitive sequence. Because about ^2^/_3_ of *RPGR* mutations reside in *ORF15_RPGR_* [37], its inaccessibility causes a diagnostic gap. Thus, male patients (but also females) without mutations in the genes investigated herein may carry mutations in *ORF15_RPGR_*. However, there was no excess of male mutation-negative RP patients: 41% of RP patients without mutations were male which corresponds to their percentage (40%) in the RP cohort (excluding the three proven X-RP patients).

The rate of mutation-negative samples sequenced on the Illumina MiSeq system was only half compared to the Roche GS FLX platform (18% versus 37%). In contrast to 454 sequencing (GS FLX), analysis of homopolymer stretches is not problematic in Solexa sequencing (MiSeq). Because very few mutations identified by supplementary Sanger sequencing (as conducted for mutation-negative LCA samples and arRP samples with monoallelic mutations) or with the Miseq were found in such sequence motifs, the higher detection rate on the MiSeq was mainly due to a better coverage in terms of completeness and depth (which allowed for CNV detection, too). As detailed above, CNVs in *PRPF31* detected by quantitative analysis of MiSeq reads resolved the diagnosis in two patients with seemingly recessive RP, and a CNV in *EYS* represented the “missing allele” in one patient.

Finally, lack of mutations may result from unclear genetic diagnosis: If an older patient is for the first time seen by an ophthalmologist at a late stage of his disease, it may be impossible to assess if the initial disease was RP or CRD; CRD genes are not comprehensively covered by our current gene panel and the causative mutation could therefore be missed.

### Comparison with Other NGS Studies on RD

This is the largest NGS study on retinal dystrophies to date. Compared to other NGS studies on this disease group, we obtained a significantly higher diagnostic yield – which is remarkable because the number of analyzed disease genes (55) in this study was much smaller than in similar studies [9], [38]–[43] (Table 2). This may in part be due to different enrichment and sequencing methods, factors that both influence depth and completeness of coverage and accuracy (for example, NGS with the 454 GS FLX platform results in a higher error rate in homopolymer stretches). High and extensive coverage, as obtained in this study, allow for systematic analysis for CNVs and reduce the risk of mutations escaping detection because of their localization in regions with low coverage. Finally, direct comparison of studies is difficult because of differences in cohort size and composition regarding phenotypes, clinical characterization and traits.

**Table 2:** Comparison of this study with previous NGS studies on retinal dystrophies.

Study	Cohort size	Genes	Platform/System	Enrichment	CNV	Phenotypes	Detection
	(no. of patients)	(n)			detection		rate
This study	126	55	GS FLX (Roche) MiSeq (Illumina)	Nimblegen (in solution)	yes	arRP	72%
						adRP	86%
						LCA	63%
						→ all	→ 70%?
Glöckle et al.	170	74	SOLiD 4, −5500×l (Life Tech)	SureSelect	no	arRP, sporadic RP	60%
[43]						adRP	41%
						LCA (4 cases)	not given
Neveling et al.	100	111	GS FLX (Roche)	Nimblegen (array)	no	RP	36%
[9]							
ÓSullivan et	50	105	SOLiD 4 (Life Tech)	SureSelect	no	RP	50–55%
al. [41]							
Shanks et al.	36*	73	GS FLX (Roche)	Nimblegen (array)	no	RP, CRD	25%
[42]							
Chen et al. [40]	25	179 (189)	GAII (Illumina)	Nimblegen	no	RP (19), STGD (2), FA (1), USH (1), BBS (1), undefined (1)	
				(array)			56%
Bowne et al.	21	46	GS FLX (Roche) GAIIx (Illumina)	PCR	no	RP (dominant)	64%
[39]							
Audo et al. [38]	17**	177 (254)	GAIIx (Illumina)	SureSelect (array)	no	RP, CSNB,	
						early CD, M. Best, STGD	57%

* Positive controls not included.

** Additional samples from the same families not included. Gene numbers in brackets include additionally screened candidate genes that are not yet proven retinal disease genes. BBS, Bardet-Biedl syndrome; CRD, cone-rod dystrophy; CD, cone dystrophy; CSNB, congenital stationary night blindness; FA, fundus albipunctatus; STGD, Morbus Stargardt; USH, Usher syndrome.

In conclusion, the identification of mutations in 28 RD genes in our cohort, with most alterations previously undescribed, clearly demonstrates that this disease group is accessible only by massively parallel multi-gene sequencing. Although our NGS study was rather conservative and confined to only 55 genes, we detected the causative mutations in the majority from a large cohort of RP and LCA patients. Regular updating of such panels and inclusion of genes for related disorders (e.g. cone-rod dystrophies) is needed to maximize the mutation detection rate. CNV detection from high-coverage NGS data was a major benefit from switching to a high-capacity NGS platform. Therefore, we currently favor NGS of an RD gene panel over exome sequencing where RD gene coverage is reduced due to distribution of reads across some 20,000 genes. Both, oligogenic heterozygosity and monoallelic constellations were observed and may require segregation analysis and careful evaluation of clinical data. Importantly, NGS readout should implicate the overall variant load in order to avoid interpretation pitfalls – as exemplified by the identification of *RP1* truncations unrelated to disease in certain constellations. “Missing alleles” in seemingly accidental carriers of recessive RD gene mutations were partly large CNVs and mutations affecting non-coding 5′ exons, demonstrating that both UTR inclusion and quantitative analysis should be part of a comprehensive NGS approach. Because such mutations may also be deep intronic variants with impact on splicing, genomic sequencing, where necessary followed by RNA analysis, may complement primary exonic sequencing in the future. Careful consideration of all variants led to revision of the assumed mode of inheritance, e.g. in case of *PRPF31* mutations in simplex RP patients.

As indicated by several exceptional findings in our study, scientific gain of knowledge will strongly benefit from the recent advent of NGS in routine diagnostics and the “byproducts” of such unprecedented large-scale analyses – not only for RD, but for many other genetically heterogeneous conditions.

## Materials and Methods

### Ethics Statement

All samples in this study were obtained with written informed consent accompanying the patients’ samples. All clinical investigations have been conducted according to the principles expressed in the Declaration of Helsinki. The study was approved by the institutional review board of the Ethics Committee of the University Hospital of Cologne.

### Patients and DNA Samples

A total of 126 patients (53 with arRP, 14 with adRP, 3 with X-RP and 56 with LCA) were included in this study. Genomic DNA was isolated from EDTA blood following standard protocols. The diagnoses of all patients were established by medical history, family history and detailed clinical evaluation of vision. Ophthalmological examination included stereoscopic funduscopy, standard ERG, perimetry, measurement of dark adaptation, and determination of best-corrected visual acuity in most patients.

### NimbleGen SeqCap EZ Choice Library Design

Genomic coordinates of coding and non-coding exons in all isoforms were identified in the RefSeq database (hg19) using the University of California Santa Cruz (USCS) table browser [44]. All coding exons (31 arRP genes, 413 exons; 23 adRP genes, 248 exons; 16 LCA genes, 215 exons) of 55 known genes (as of end of year 2010; Table S1 in File S1) including 35 bp of flanking 5′ and 3′ intronic sequence were targeted by a custom SeqCap EZ Choice library (NimbleGen, Madison, Wisconsin, USA). In total, 752 regions were targeted comprising 213 kb of target sequence. The final design covered about 99% of the requested target regions. Because of its highly repetitive sequence which precludes efficient enrichment and sequencing, *RPGR* exon *ORF15* was excluded from panel design. Because the *USH2A* gene was not included in the panel design, all coding exons of the gene were analyzed either by conventional Sanger sequencing or by a complementary *USH2A*-including NGS gene panel in arRP patients without mutations in the RP genes covered by our panel.

### Sequence Capture and Next-generation Sequencing (NGS)

Samples from 79 patients were subjected to NGS on the Roche GS FLX platform (454 Life Sciences, Branford, CT; average output 400–500 Mb). In the second part of the study, 38 samples were sequenced on the Illumina MiSeq system (Illumina, San Diego, CA; average output 1,5–5 Gb) with only the latter allowing for CNV detection due to high and uniform coverage. Samples from nine patients were analysed on both systems (two samples with no mutations, four samples with monoallelic mutations in 454 sequencing and three samples with confirmed mutations from 454 sequencing). Between eight (GS FLX) and 20 (MiSeq) samples were pooled and sequenced in a multiplexing procedure. Multiple DNAs were enriched using the NimbleGen SeqCap EZ choice sequence capture approach and sequenced by Roche 454 GS FLX pyrosequencing or by Illumina MiSeq sequencing-by-synthesis technology according to the manufacturerś protocols. In brief, 0.5–1 µg of genomic DNA per sample was sheared using the Covaris S2 AFA system (Covaris Inc., Woburn, MA, USA) and ligated to barcoded adaptors for multiplexing. Pre-capture amplified samples were pooled and hybridized to the customized in-solution capture library for 72 hours, subsequently eluted and post-capture amplified by ligation-mediated (LM-) PCR. This amplified enriched DNA was used as input for emulsion PCR (emPCR) and subsequent massively parallel sequencing on one full PTP of a Roche 454 GS FLX platform or as input for direct cluster generation and sequencing on the Illumina MiSeq system (2×150 bp paired-end reads). Uncovered regions of LCA genes (n = 16) in negative samples from LCA patients designated as having “LCA” were sequenced by conventional Sanger sequencing for completeness, whereas in RP samples, gaps of uncovered exons of arRP genes (n = 31) samples were only eliminated by Sanger sequencing in search of a second mutation in an incompletely covered arRP gene).

### Read Mapping and Variant Analysis

Demultiplexed reads from the GS FLX platform or paired end reads (2×150 bp) from the Illumina MiSeq instrument were mapped against the hg19 human reference genome using SMALT (Ponstingl and Ning, 2010, Wellcome Trust Sanger Institute) with the recommended standard settings. The mapped reads were preprocessed with SAMtools [45] and duplicate reads were marked by Picard (http://picard.sourceforge.net). Finally, GATK [46] was applied for a local realignment and base quality score recalibration of the mapped reads. Mapping and coverage statistics were generated from the mapping output files using the SeqCap analysis toolkit provided by Roche 454 as well as GATK. Identified variants were checked against the dbNSFP v1.3 [47] as well as dbSNP v135 and HGMD® Professional 2011.4 database (released December 9, 2011). SNVs and indels were filtered depending on their allele frequency focusing on rare variants with a minor allele frequency (MAF) of 3% or less. Nonsense, frameshift and canonical splice site variants were considered pathogenic. Pathogenicity of a rare non-synonymous single nucleotide variations (nSNVs) scores of which were not yet predicted in dbNSFP were assessed using five *in silico* prediction software tools: SIFT [48], Mutation Taster [49], PolyPhen-2 [50], AlignGVGD [51], [52] and PMut [53]. An nSNV was considered likely pathogenic when at least three of these algorithms predicted that the variant is probably damaging and when it was predicted as conserved with the conservation prediction algorithms PhyloP [54] and GERP++ [55]. The impact of splice site variants was assessed using splice site prediction programmes NNSPLICE v0.9 [56], NetGene2 [57], [58], SpliceView [59] and ESEfinder [60]. Variants not listed in HGMD [61] were considered novel. For visualization of the identified SNVs, SFF files (Roche 454) or FASTQ files (Illumina) of the patients’ sample were loaded into the SeqPilot SeqNext module (v4.0, JSI medical systems, Kippenheim, Germany), and reads were mapped against the genomic sequences of the genes in the indicated subpanels arRP, adRP or LCA. SNVs were filtered by their occurrence in at least 25% of the reads. Distinct variations were checked against the in-house database. Due to inaccurate sequencing of homopolymers by Roche 454 pyrosequencing, small indels in homopolymer stretches were filtered using stringent criteria (bidirectional occurrence in at least 20% of the forward reads and 40% of the reverse reads or vice versa) and visual inspection in the SeqNext software. Identified sequence variants were annotated according to the guidelines published by the Human Genome Variation Society.

### Validation and Segregation Analysis

Sequence variants of interest identified by high-throughput sequencing were verified by Sanger sequencing following PCR amplification of the respective coding exons and adjacent intronic sequences by standard protocols. Purified PCR fragments were sequenced using Big Dye Terminator Cycle sequencing and analyzed on an 3500 Genetic Analyzer sequencer (Applied Biosystems, Foster City, CA, USA). Where applicable, DNA from affected and unaffected family members was analyzed for segregation analysis of putatively causative sequence variants. The resulting sequence data were compared to the reference sequence of the RefSeq database [62].

### Copy Number Variation Analysis

Very high coverage was reproducibly achievable by sequencing with the Illumina MiSeq system and enabled copy number variation (CNV) analysis for most of the analyzed genes. Potential copy number alterations (CNA) were initially identified by VarScan [63] on mapped reads. Thereby, coverage of every target region of the sample of interest was internally normalized and compared versus normalized control data of other samples of the same run (VarScan copy number mode and standard settings). Potential CNVs were reported, if the CNV was detected against at least 75% of the control patients. CNVs were annotated using refGene from UCSC (ftp://hgdownload.cse.ucsc.edu/goldenPath/hg19/database/refGene.txt.gz). Potential CNVs were visualized and recalculated with the CNV mode of SeqNext using standard settings and the analysis mode “all vs. all.” Thereby, the normalized relative coverage of every target ROI (region of interest) of a patient sample (relative product coverage, RPC P.) was calculated against the normalized average relative target coverage of several control samples (RPC C.) to obtain the ratio relative coverage (ratio RPC). Deletions were reported if the ratio RPC fell below 75%. CNVs that fulfilled these criteria were validated by multiplex ligation dependent probe amplification (MLPA) for the affected gene. For the *EYS* gene, the SALSA MLPA probemix P-328-A1 *EYS*, for the *CRX* gene the SALSA MLPA probemix P221-B1 LCA and for the *PRPF31* gene the SALSA MLPA KIT P235-B1 Retinitis Pigmentosa was used (MRC-Holland, Amsterdam, The Netherlands). Only CNVs that could be confirmed by MLPA were considered real. MLPA results were visualized with the MLPA module of the SeqPilot software (JSI Medical Systems). The ratio RPA (relative peak area) was calculated as the RPA of the patient versus controls.

### Exclusion of the *CEP290* Hot Spot Mutation in LCA Patients

For exclusion of the common c.2991+1655A>G mutation in the *CEP290* gene mutation in all LCA patients prior to NGS analysis, the region of interest in intron 26 was amplified by PCR. Genotyping for the presence of the mutation was performed by pyrosequencing using QIAGEN Pyro Gold chemistry according to the manufactureŕs instructions and subsequent analysis on a PSQ 96MA system (QIAGEN, Hilden, Germany).

### Linkage Analysis

In the family of patient 110 afflicted with LCA, we performed genome-wide homozygosity mapping using the Affymetrix GeneChip Human Mapping 10K Array, version 2.0 (Affymetrix, Santa Clara, CA). GRR [64] and PedCheck [65] were used to verify relationships and to identify Mendelian errors. Nonparametric linkage analysis was done with MERLIN [66]. Parametric linkage and haplotype analysis was performed using the program ALLEGRO [67]. All data handling was performed using the graphical user interface ALOHOMORA [68]. Graphic output of haplotypes was generated with HaploPainter [69].

## Supporting Information

File S1 **File S1 contains the following files.** **Figure S1.** **CNVs (from partial to complete gene deletions) of** ***PRPF31*** **detected by analysis of NGS data.** A heterozygous deletion of all 14 *PRPF31* exons was identified in patient 113. In patient 116, exons 1–5 were deleted on one gene copy (the non-coding exon 1 was not yet included in target enrichment and subsequent NGS, but its deletion was confirmed by MLPA in both patients). The dashed line and red arrows indicate lower coverage for heterozygously deleted regions compared to one control sample. **Figure S2. Validation of CNVs predicted from NGS data by MLPA.** Only confirmed CNVs were considered true CNVs. **A.** Heterozygous deletion of exon 1 in the *EYS* gene in patient 57 and his father. **B.** Heterozygous deletion of exon 4 in the *CRX* gene in patient 110 and his father. **C.** Heterozygous deletion of exons 1–14 in the *PRPF31* gene in patient 113 and of exons 1–5 in patient 116. RPA: Relative peak area of the patient result file (green) and of the control result files (blue) with standard deviation (error bar). The ratio RPA was calculated as the RPA of the patient versus controls. Deletions are indicated if the ratio RPA falls below 75%. **Table S1. Genes analyzed in this study. A.** arRP, adRP and LCA genes that were captured and subjected to NGS in this study. **B.** Functional categorization of genes with causative mutations. **Table S2. Additional variants classified as “likely pathogenic”.** Classification as pathogenic by at least three out of five bioinformatic prediction programs and a minor allele frequency below 3% in unresolved patients. Although a contribution of these variants to the phenotype cannot be excluded, they were not considered causative. In many cases, they represented monoallelic variants in recessive genes which would not sufficiently explain the phenotype. **References S1. References for** **Table 1** **and Table S2.**
(ZIP)
