# Correction: Increasing The Yield In Targeted Next-Generation Sequencing By Implicating Cnv Analysis, Non-Coding Exons And The Overall Variant Load: The Example Of Retinal Dystrophies

**DOI:** 10.1371/journal.pone.0108840

**Published:** 2014-11-13

**Authors:** The PLOS ONE Staff

There is an error in the header of Table 1. The row “Autosomal recessive retinitis pigmentosa, arRP” should not be a separate header for the entire table. The publisher apologizes for this error. A corrected version of Table 1 can be viewed below.

**Table 1:** Causative mutations and putatively pathogenic variants identified in this study.

Patient	Gene	Allele 1				Allele 2				Additional Allele				Gender	Age (years)	Phenotype	Inheritance (family history)	Consang.	Origin
		cds	Protein	db SNP	Ref	cds	Protein	dbSNP	Ref	cds	Protein	dbSNP	Ref						
**Autosomal recessive retinitis pigmentosa, arRP**																			
2	*ABCA4*	c.768G>T	p.V256V	-	[1,2,3]	c.5603A>T	p.Asn1868Ile	rs1801466	[4,5]					m	60	RP	ar/s	no	Cau
			(splice)																
30	*ABCA4*	c.1622T>C/	p.Leu541Pro/	rs61751392		c.3210_3211	p.Ser1071Cys			*ABCA4:*				f	17	RP	ar/s	no	Ger
		c.3113C>T	p.Ala1038Val	rs61751374	[6,7,8]	dupGT	fs*14	-	[9]	c.5603A>T	p.Asn1868Ile	rs1801466	[4,5]						
31	*ABCA4*	c.768G>T	p.V256V	-	[1,2,3]	c.1622T>C/	p.Leu541Pro/	rs61751392	[6,7,8]					f	8	RP	ar/s	no	Ger
			(splice)			c.3113C>T	p.Ala1038Val	rs61751374											
53	*ABCA4*	c.1A>G	p.Met1Val	-	[10]	c.1A>G	p.Met1Val	-	[10]	*ABCA4:*				f	44	RP	ar/s	yes	Ger
			(p.Met1?)				(p.Met1?)			c.6089G>A	p.Arg2030Gln	rs61750641	[10,11]						
										c.6089G>A	p.Arg2030Gln	rs61750641	[10,11]						
3	*C2ORF71*	c.2756_2768del	p.Lys919Thr	-	[12]	c.2756_2768del	p.Lys919Thr	-	[12]					f	44	RP	ar/s	yes	Tur
			fs*2				fs*2												
45	*CNGA1*	c.1036C>T	p.Arg346Trp	-	[a]	c.1166C>T	p.Ser389Phe	rs62625014	[13]	*PDE6B:*				m	43	RP	ar/s	no	Ger
										c.703C>T	p.Arg235Cys	-	[a]						
74	*CNGA1*	c.2195A>G	p.Glu732Gly	-	[a]	c.2195A>G	p.Glu732Gly	-	[a]	*TTC8*:				m	50	RP	ar/s	no	Ger
										c.1253A>G	p.Gln418Arg	rs142938748	[a]						
5	*CRB1*	c.1459T>C	p.Ser487Pro	-	[a]	c.1459T>C	p.Ser487Pro	-	[a]	*SAG:*				m	n.d.	RP	ar/s	yes	Pak
										c.374C>T	p.Thr125Met	rs137886124	[a]						
7	*CRB1*	c.2042G>A	p.Cys681Tyr	rs62636266	[14]	c.2308G>C	p.Gly770Arg	-	[a]	*FSCN2:*				f	28	RP	ar/s	no	Pol
										c.805C>T	p.Arg269Cys	-	[a]						
48	*CRB1*	c.2367T>A	p.Asn789Lys	-	[a]	c.2401A>T	p.Lys801*	-	[15]					f	37	RP	ar/s	no	Ger
8	*EYS*	c.604T>C	p.Cys202Arg	-	[a]	c.4350_4356del	p.Ile1451Pro	-	[16]	*MERTK:*				m	52	RP	ar/s	no	Au
							fs*3			c.791C>G	p.Ala264Gly	-	[a]						
										*SAG:*									
										c.473C>A	p.Thr158Lys	-	[a]						
34	*EYS*	c.7055+1G>T	splice	-	[a]	c.7055+1G>T	splice	-	[a]					f	40	RP	ar/s	yes	Syr
57	*EYS*	c.4045C>T	p.Arg1349*	-	[a]	deletion of	?	-	[a]					f	32	RP	ar/s	no	Ger
						exon 1													
88	*EYS*	c.67delA	p.Thr23His	-	[a]	c.162C>A	p.Tyr54*	-	[a]					m	48	RP	ar/Xl	no	Ger
			fs*19																
93	*EYS*	c.4350_4356del	p.Ile1451Pro	-	[16]	c.-448+5G>A	splice	-	[a]	*RD3:*				m	23	RP	ar/s	no	Ger
			fs*3							c.584A>T	p.Asp195Val	rs143207434	[a]						
9	*MERTK*	c.345C>G	p.Cys115Trp	-	[a]	c.1530delT	p.Cys510Trp	-	[a]					m	19	RP	ar/Xl	no	Ger
							fs*5												
46	*MERTK*	c.1786G>A	p.Gly596Arg	-	[a]	c.1786G>A	p.Gly596Arg	-	[a]					f	36	RP	ar/s	n.d.	n.d.
76	*MERTK*	c.1450G>A	p.Gly484Ser	-	[a]	c.1450G>A	p.Gly484Ser	-	[a]	*RP2:*				m	30	RP	ar/s	no	Italy
										c.844C>T	p.Arg282Trp	rs1805147	[17,18]						
										*RHO:*									
										c.310G>A	p.Val104Ile	rs144317206	[19]						
20	*PDE6B*	c.669T>A	p.Tyr223*	-	[a]	c.669T>A	p.Tyr223*	-	[a]	*RBP3:*				f	30	RP	ar/s	yes	Iran
										c.2092C>A	p.Arg698Ser	-	[a]						
21	*PDE6B*	c.1699C>T	p.Gln567*	-	[a]	c.1699C>T	p.Gln567*	-	[a]	*ABCA4:*				f	36	RP	ar/s	yes	Ger
										c.5603A>T	p.Asn1868Ile	rs1801466	[4,5]						
62	*PDE6B*	c.2193+1G>A	splice	-	[20]	c.2193+1G>A	splice	-	[20]	*ABCA4*:				m	47	RP	ar/s	yes	Ger
										c.2184C>A	p.Ser728Arg	-	[a]						
75	*PDE6B*	c.2193+1G>A	splice	-	[20]	c.2047G>A	p.Val683Met	-	[a]	*PDE6B:*				m	45	RP	ar/s	no	Ger
										c.2249T>G	p.Val750Gly	-	[a]						
55	*PROM1*	c.642T>A	p.Tyr214*	-	[a]	c.1209_1229del	p.Gln403_Ser	-	[a]	*RP1:*				m	26	RP	ar/s	no	Ger
							410delinsHis			c.6304C>T	p.Gln2102*	-	[a]						
										*CRB1:*									
										c.2042G>A	p.Cys681Tyr	rs62636266	[14]						
										*RPGRIP1:*									
										c.1767G>T	p.Gln589His	rs34067949	[21,22]						
89	*RDH12*	c.226G>C	p.Gly76Arg	-	[23]	c.869T>G	p.Val290Gly	rs61740289	[a]	*ABCA4:*				f	34	RP	ar/s	yes	DRC
										c.618C>G	p.Ser206Arg	rs61748536	[24]						
										*C2ORF71*:									
										c.1844T>A	p.Val615Asp	rs140776870	[25]						
25	*RP1*	c.597C>A	p.Tyr199*	-	[a]	c.3157delT	p.Tyr1053Thr	-	[26]	*CDH23:*				f	38	RP	ar/s	no	Ger
							fs*4			c.6322G>T	p.Glu2108*	-	[27]						
										*CRB1:*									
										c.3122T>C	p.Met1041Thr	rs62635656	[28]						
26	*RP1*	c.4242_4243del	p.His1414Gln	-	[a]	c.4474G>T	p.Glu1492*	-	[a]					f	47	RP	ar/s	yes	Iran
			fs*5																
27	*RP1*	c.1012C>T	p.Arg338*	-	[a]	c.1012C>T	p.Arg338*	-	[a]	*ABCA4:*				f	37	RP	ar/s	yes	Iran
										c.5603A>T	p.Asn1868Ile	rs1801466	[4,5]						
28	*RP1*	c.5278_5287del	p.Asn1760Cys	-	[a]	c.5278_5287del	p.Asn1760Cys	-	[a]					f	12	RP	ar/s	yes	Tur
			fs*46				fs*46												
36	*RP1*	c.607G>A	p.Gly203Arg	-	[a]	c.607G>A	p.Gly203Arg	-	[a]					m	n.d.	RP	ar/s	yes	Iran
101	*RP1*	c.3843delT	p.Pro1282Leu	-	[a]	c.3843delT	p.Pro1282Leu	-	[a]					f	37	RP	ar/s	yes	Tur
			fs*12				fs*12												
29	*SAG*	c.577C>T	p.Arg193*	-	[29]	**?**								m	16	RP	ar/s	no	Ger
33	*TULP1*	c.371_394del	p.Asp124_	-	[a]	**?**				*CRX:*				m	52	RP	ar/s	no	Ger
			132delinsAla							c.122G>A	p.Arg41Gln	rs61748436	[30]						
										*CRX:*									
										c.425A>G	p.Tyr142Cys	rs61748442	[21]						
82	*TULP1*	c.371_394del	p.Asp124_	-	[a]	**?**								f	35	RP	ar/s	no	Ger
			132delinsAla																
37	*TULP1*	c.1047T>G	p.Asn349Lys	-	[a]	c.1047T>G	p.Asn349Lys	-	[a]	*PDE6A:*				f	n.d.	RP	ar/s	yes	Iran
										c.923C>T	p.Pro308Leu	-	[a]						
38	*TULP1*	c.1198C>T	pArg400Trp	-	[31]	c.1198C>T	pArg400Trp	-	[31]	*ABCA4:*				f	n.d.	RP	ar/s	yes	Iran
										c.2353C>T	p.Arg785Cys	-	[a]						
										*FSCN2:*									
										c.325C>T	p.Arg109Cys	-	[a]						
13	*USH2A*	c.10421A>G	p.Tyr3474Cys	-	[a]	c.13257_13263	p.Phe4419Leu	-	[a]					m	26	RP	ar/s	no	Ger
						del	fs*2												
18	*USH2A*	c.6925T>C	p.Cys2309Arg	-	[a]	c.10561T>C	p.Trp3521Arg	rs111033264	[32,33]	*ABCA4:*				f	50	RP	ar/s	no	Ger
										c.5603A>T	p.Asn1868Ile	rs1801466	[4,5]						
										*AIPL1:*									
										c.401A>T	p.Tyr134Phe	rs16955851	[34]						
68	*USH2A*	c.1256G>T	p.Cys419Phe	rs121912600	[35]	c.10342G>A	p.Glu3448Lys	-	[a]					m	44	RP	ar/s	no	Ger
**Autosomal dominant RP, adRP**																			
22	*PRPF31*	c.1048C>T	p.Gln350*	-	[a]					*RPE65:*				f	38	RP	ar/s	no	E-Eur
										c.963T>G	p.Asn321Lys	rs149916178	[36]						
23	*PRPF31*	c.1067_1073+8	Splice	-	[a]									f	36	RP	ar/s	no	Ger
		del																	
43	*PRPF31*	c.217A>T	p.Lys73*	-	[a]									m	18	RP	ad	no	Ger
113	*PRPF31*	deletion of	haplo-	-	[37,38]					*ABCA4:*				f	55	RP	ad	no	Ger
		exons 1-14	insufficiency							c.1928G>A	p.Val643Gly	rs114572202	[39]						
										c.5603A>T	p.Asn1868Ile	rs1801466	[4,5]						
										*RPGRIP1:*									
										c.2555G>A	p.Arg852Gln	rs181758389	[21]						
116	*PRPF31*	deletion of	haplo-	-	[37,38]									f	14	RP	ar/s	No	Cau
		exons 1-5	insufficiency																
47	*PRPH2*	c.920delT	p.Leu307Arg	-	[40]					*FSCN2*:				m	33	RP	ad	no	Ger
			fs*17							c.412C>T	p.His138Tyr	rs143796236	[a]						
32	*RHO*	c.35C>G	p.Pro12Arg	-	[a]					*RLBP1:*				f	71	RP	ad	no	Ger
										c.545T>G	p.Phe182Cys	rs142244640	[a]						
44	*RHO*	c.541G>A	p.Glu181Lys	-	[41]									f	47	RP	s	no	Ger
92	*RHO*	c.180C>A	p.Tyr60*	-	[a]					*ROM1:*				f	31	RP	ad	no	Ger
										c.178C>A	p.Pro60Thr;	-	[42,43]						
										c.323C>T	p.Thr108Met	rs146358003	[42,43]						
										*ABCA4:*									
										c.1654G>A	p.Val552Ile	rs145525174	[44]						
										c.5714+5G>A	splice	rs61751407	[45]						
										*RP2:*									
										c.844C>T	p.Arg282Trp	rs1805147	[17,18]						
										*RPGRIP1:*									
										c.2510C>G	p.Ala837Gly	-	[22]						
121	*RHO*	c.937-1G>T	splice	-	[46,47]					*ABCA4:*				m	25	RP	ad	no	Cau
										c.5603A>T	p.Asn1868Ile	rs1801466	[4,5]						
103	*TOPORS*	c.2554_2557del	p.Glu852Gln	-	[a]									f	30	RP	ad	no	Ger
			fs*13																
108	*TOPORS*	c.2550_2553del	p.Asp850Glu	-	[a]					*ABCA4:*				f	59	RP	ad	no	Ger
			fs*15							c.4771G>A	p.Gly1591Arg	rs113106943	[a]						
										*PDE6A:*									
										c.298C>T	p.Arg100Trp	-	[a]						
**X-linked RP**																			
77	*RP2*	c.226G>T	p.Asp76Tyr	-	[a]					*RP2:*				m	15	RP	ar/s	no	SE-Eur
										c.844C>T	p.Arg282Trp	rs1805147	[17,18]						
										*ABCA4:*									
										c.5882G>A	p.Gly1961Glu	rs1800553	[7,48]						
100	*RPGR*	c.1853_1856dup	p.Glu621Lys	-	[a]									f	54	RP	ar	no	Ger
			fs*10																
119	*RPGR*	c.1544T>G	p.Leu515*	-	[a]									m	5	LCA	ar	no	KSA
**Leber congenital amaurosis, LCA**																			
1	*AIPL1*	c.834G>A	p.Trp278*	rs62637014	[49]	c.834G>A	p.Trp278*	rs62637014	[49]	*RD3:*				m	2	LCA	ar	possible	Ger
										c.584A>T	p.Asp195Val	rs143207434	[a]						
										*SAG:*									
										c.374C>T	p.Thr125Met	rs137886124	[a]						
83	*AIPL1*	c.50T>C	p.Leu17Pro	-	[50]	c.50T>C	p.Leu17Pro	-	[50]	*CRB1:*				f	10	LCA	ar	possible	Tur
										c.3397G>A	p.Val1133Met	-	[a]						
										*SEMA4A:*									
										c.1301T>C	p.Met434Thr	rs146822426	[a]						
4	*CEP290*	c.3640dupG	p.Glu1214Gly	-	[a]	c.6604delA	p.Ile2202Leu	-	[51]					f	2	LCA	ar	no	Au
			fs*7				fs*24												
80	*CEP290*	c.2578G>T	p.Glu860*	-	[a]	c.3758G>A	p.Arg1253His	-	[a]	*CEP290:*				f	9	LCA (early CRD)	ar	no	Tur
										c.5254C>T	p.Arg1752Trp	-	[a]						
										*FSCN2:*									
										c.377C>T	p.Ser126Phe	-	[a]						
94	*CEP290*	c.6012-2A>G	splice	-	[a]	c.6870delT	p.Gln2291Lys	-	[52]	*ABCA4:*				f	5	LCA	ar	no	KSA
							fs*10			c.1140T>A;	p.Asn380Lys	rs61748549	[4]						
										c.5642C>T;	p.Ala1881Val	-	[53]						
										c.5882G>A	p.Gly1961Glu	rs1800553	[7,48]						
										*RP1:*									
										c.1380G>C	p.Lys460Asn	rs143494598	[a]						
6	*CRB1*	c.2842+2T>A	splice	-	[a]	c.2842+2T>A	splice	-	[a]					m	20	LCA		no	Tur
63	*CRB1*	c.1180T>C	p.Cys394Arg	-	[a]	c.1180T>C	p.Cys394Arg	-	[a]					m	6	LCA	ar	yes	KSA
110	*CRX*	deletion of exon 4	NMD?	-	[a]	c.899A>G	p.(*300Trp	-	[a]	*RP1:*				f	9	LCA	ar	yes	Tur
							ext*118)			c.3191G>T	p.Ser1064Ile	-	[a]						
										*SPATA7:*									
										c.1112T>C	p.Ile371Thr	rs150364664	[a]						
56	*GUCY2D*	c.1093_1106del	p.Arg365Trp	-	[a]	c.1093_1106del	p.Arg365Trp	-	[a]					m	4	LCA	ar	yes	Tur
			fs*77				fs*77												
71	*GUCY2D*	c.1401dupT	p.Leu468Ser	-	[a]	c.1401dupT	p.Leu468Ser	-	[a]	*RDH12:*				m	5	LCA	ar	n.d.	KSA
			fs*89				fs*89			c.917T>G	p.Val306Gly	-	[a]						
73	*GUCY2D*	c.2766C>G	p.Tyr922*	-	[a]	c.2766C>G	p.Tyr922*	-	[a]	*GUCY2D:*				f	18	LCA	ar	yes	Tur
										c.2927G>T	p.Arg976Leu	rs61750184	[54]						
										c.2927G>T	p.Arg976Leu	rs61750184	[54]						
118	*GUCY2D*	c.2080C>T	p.Gln694*	-	[a]	?								m	5	LCA	ar	no	Ger
123	*GUCY2D*	c.389delC	p.Pro130Leu	rs61749670	[55]	c.389delC	p.Pro130Leu	rs61749670	[55]					f	1	LCA	ar	no	Mor
			fs*36				fs*36												
125	*GUCY2D*	c.1401dupT	p.Leu468Ser	-	[a]	c.1401dupT	p.Leu468Ser	-	[a]	*RDH12:*			[a]	f	5	LCA	ar	yes	KSA
			fs*89				fs*89			c.917T>G	p.Val306Gly	-							
107	*LCA5*	c.763C>T	p.Arg255*	rs151017794	[a]	c.763C>T	p.Arg255*	rs151017794	[a]	*ABCA4*:				f	1	LCA	ar	n.d.	KSA
										c.1927G>A	p.Val643Met	rs61749417	[10]						
										*PDE6B:*									
										c.704G>A	p.Arg235His	-	[a]						
84	*LRAT*	c.233_242del	p.Leu78Arg	-	[a]	c.233_242del	p.Leu78Arg	-	[a]					m	19	LCA	ar	yes	KSA
			fs*85				fs*85												
87	*LRAT*	c.449dupG	p.Phe151Leu	-	[a]	c.449dupG	p.Phe151Leu	-	[a]					f	11	LCA	ar	yes	Tur
			fs*33				fs*33												
70	*MERTK*	c.1744_1751	p.Ile582*	-	[a]	c.1744_1751	p.Ile582*	-	[a]	*RBP3:*				m	16	LCA	ar	yes	Tur
		delinsT				delinsT				c.2789T>C	p.Ile930Thr	-	[a]						
24	*RDH5*	c.602C>T	p.Ser201Phe	-	[a]	c.602C>T	p.Ser201Phe	-	[a]					f	15	LCA (early CRD)	ar	yes	Pak
49	*RDH12*	c.133A>G	p.Thr45Ala	-	[56]	c.133A>G	p.Thr45Ala	-	[56]	*GUCY2D:*				m	12	LCA	ar	no	Ger
										c.2359T>G	p.Cys787Gly	-	[a]						
										*SPATA7:*									
										c.3G>A	p.Met1Ile (p.Met1?)	-	[57]						
120	*RDH12*	c.188G>T	p.Gly63Val	-	[a]	c.188G>T	p.Gly63Val	-	[a]	*ABCA4:*			[a]	m	7	LCA	ar	yes	KSA
			(splice)				(splice)			c.3482G>T	p.Arg1161Leu	-							
95	*RPE65*	c.271C>T	p.Arg91Trp	rs61752871	[58]	c.271C>T	p.Arg91Trp	rs61752871	[58]	*CDHR1:*				f	28	LCA	ar	n.d.	KSA
										c.1202G>C,	p.Gly401Ala,	-	[a]						
										c.1202G>C	p.Gly401Ala;	-	[a]						
										*LCA5:*									
										c.764G>A	p.Arg255Gln	-	[a]						
35	*RPGRIP1*	c.2608_2609insA	p.Leu870Tyr	-	[a]	c.2608_2609insA	p.Leu870Tyr	-	[a]	*RPE65:*				f	9	LCA	ar	no	Dubai
			fs*7				fs*7			c.394G>A	p.Ala132Thr	rs61752878	[a]						
50	*RPGRIP1*	c.1107delA	p.Glu370Asn	rs61751266	[59]	c.1107delA	p.Glu370Asn	rs61751266	[59]					m	6	LCA	ar	yes	KSA
			fs*5				fs*5												
81	*RPGRIP1*	c.3565C>T	p.Arg1189*	-	[a]	c.3565C>T	p.Arg1189*	-	[a]	*TTC8:*				m	11	LCA	ar	n.d.	KSA
										c.512A>T	p.Asp171Val	-	[a]						
90	*RPGRIP1*	c.1107delA	p.Glu370Asn	rs61751266	[59]	c.1107delA	p.Glu370Asn	rs61751266	[59]	*MERTK:*				m	1	LCA	ar	n.d.	KSA
			fs*5				fs*5			c.2435A>C	p.Tyr812Ser	rs141361084	[a]						
109	*RPGRIP1*	c.2662C>T	p.Arg888*	-	[a]	c.2662C>T	p.Arg888*	-	[a]	*C2ORF71*:				f	6	LCA	ar	yes	KSA
										c.1844T>A	p.Val615Asp	rs140776870	[25]						
111	*RPGRIP1*	c.1107delA	p.Glu370Asn	rs61751266	[59]	c.1107delA	p.Glu370Asn	rs61751266	[59]	*RBP3:*				f	6	LCA	ar	yes	KSA
			fs*5				fs*5			c.490G>A	p.Ala164Thr	-	[a]						
115	*RPGRIP1*	c.1107delA	p.Glu370Asn	rs61751266	[59]	c.1107delA	p.Glu370Asn	rs61751266	[59]	*PROM1:*				f	2	LCA	ar	yes	KSA
			fs*5				fs*5			c.2094C>A	p.Ser698Arg	-	[a]						
96	*TULP1*	c.901C>T	p.Gln301*	-	[60]	c.901C>T	p.Gln301*	-	[60]	*C2ORF71*:				m	7	LCA	ar	yes	KSA
										c.1844T>A	p.Val615Asp	rs140776870	[25]						
112	*TULP1*	c.901C>T	p.Gln301*	-	[60]	c.901C>T	p.Gln301*	-	[60]					m	19	LCA	ar	no	KSA
114	*TULP1*	c.1604T>C	p.Phe535Ser	-	[a]	c.1604T>C	p.Phe535Ser	-	[a]					f	7	LCA	ar	n.d.	UEA
117	*TULP1*	c.901C>T	p.Gln301*	-	[60]	c.901C>T	p.Gln301*	-	[60]					f	8	LCA	ar	yes	KSA
122	*TULP1*	c.901C>T	p.Gln301*	-	[60]	c.901C>T	p.Gln301*	-	[60]					f	3	LCA	ar	yes	KSA
124	*TULP1*	c.901C>T	p.Gln301*	-	[60]	c.901C>T	p.Gln301*	-	[60]	*RP1:*				m	6	LCA	ar	possible	KSA
										c.5248G>T	p.Glu1750*	-	[a]						

doi:10.1371/journal.pone.0078496.t001
