# Supplementary material for: Increasing the Yield in Targeted Next-Generation Sequencing by Implicating CNV Analysis, Non-Coding Exons and the Overall Variant Load: The Example of Retinal Dystrophies
Source: PLoS One. 2013 Nov 12;8(11):e78496. doi: 10.1371/journal.pone.0078496 (PMC3827063; doi:10.1371/journal.pone.0078496)
Supplement: File S1 — contains the following files. Figure S1. CNVs (from partial to complete gene deletions) of PRPF31 detected by analysis of NGS data. A heterozygous deletion of all 14 PRPF31 exons was identified in patient 113. In patient 116, exons 1–5 were deleted on one gene copy (the non-coding exon 1 was not yet included in target enrichment and subsequent NGS, but its deletion was confirmed by MLPA in both patients). The dashed line and red arrows indicate lower coverage for heterozygously deleted regions compared to one control sample. Figure S2. Validation of CNVs predicted from NGS data by MLPA. Only confirmed CNVs were considered true CNVs. A. Heterozygous deletion of exon 1 in the EYS gene in patient 57 and his father. B. Heterozygous deletion of exon 4 in the CRX gene in patient 110 and his father. C. Heterozygous deletion of exons 1–14 in the PRPF31 gene in patient 113 and of exons 1–5 in patient 116. RPA: Relative peak area of the patient result file (green) and of the control result files (blue) with standard deviation (error bar). The ratio RPA was calculated as the RPA of the patient versus controls. Deletions are indicated if the ratio RPA falls below 75%. Table S1. Genes analyzed in this study. A. arRP, adRP and LCA genes that were captured and subjected to NGS in this study. B. Functional categorization of genes with causative mutations. Table S2. Additional variants classified as “likely pathogenic”. Classification as pathogenic by at least three out of five bioinformatic prediction programs and a minor allele frequency below 3% in unresolved patients. Although a contribution of these variants to the phenotype cannot be excluded, they were not considered causative. In many cases, they represented monoallelic variants in recessive genes which would not sufficiently explain the phenotype. References S1. References for Table 1 and Table S2. (ZIP) [file pone.0078496.s001.zip › Additional Data Files/References S1.doc]

1. Maugeri A, van Driel MA, van de Pol DJ, Klevering BJ, van Haren FJ, Tijmes N, Bergen AA, Rohrschneider K, Blankenagel A, Pinckers AJ *et al*: **The 2588G-->C mutation in the ABCR gene is a mild frequent founder mutation in the Western European population and allows the classification of ABCR mutations in patients with Stargardt disease**. *Am J Hum Genet* 1999, **64**(4):1024-1035.

2. Klevering BJ, Maugeri A, Wagner A, Go SL, Vink C, Cremers FP, Hoyng CB: **Three families displaying the combination of Stargardt's disease with cone-rod dystrophy or retinitis pigmentosa**. *Ophthalmology* 2004, **111**(3):546-553.

3. Collin RW, van den Born LI, Klevering BJ, de Castro-Miro M, Littink KW, Arimadyo K, Azam M, Yazar V, Zonneveld MN, Paun CC *et al*: **High-resolution homozygosity mapping is a powerful tool to detect novel mutations causative of autosomal recessive RP in the Dutch population**. *Invest Ophthalmol Vis Sci* 2011, **52**(5):2227-2239.

4. Webster AR, Heon E, Lotery AJ, Vandenburgh K, Casavant TL, Oh KT, Beck G, Fishman GA, Lam BL, Levin A *et al*: **An analysis of allelic variation in the ABCA4 gene**. *Invest Ophthalmol Vis Sci* 2001, **42**(6):1179-1189.

5. Aguirre-Lamban J, Gonzalez-Aguilera JJ, Riveiro-Alvarez R, Cantalapiedra D, Avila-Fernandez A, Villaverde-Montero C, Corton M, Blanco-Kelly F, Garcia-Sandoval B, Ayuso C: **Further associations between mutations and polymorphisms in the ABCA4 gene: clinical implication of allelic variants and their role as protector/risk factors**. *Invest Ophthalmol Vis Sci* 2011, **52**(9):6206-6212.

6. Rivera A, White K, Stohr H, Steiner K, Hemmrich N, Grimm T, Jurklies B, Lorenz B, Scholl HP, Apfelstedt-Sylla E *et al*: **A comprehensive survey of sequence variation in the ABCA4 (ABCR) gene in Stargardt disease and age-related macular degeneration**. *Am J Hum Genet* 2000, **67**(4):800-813.

7. Cella W, Greenstein VC, Zernant-Rajang J, Smith TR, Barile G, Allikmets R, Tsang SH: **G1961E mutant allele in the Stargardt disease gene ABCA4 causes bull's eye maculopathy**. *Exp Eye Res* 2009, **89**(1):16-24.

8. Cideciyan AV, Swider M, Aleman TS, Tsybovsky Y, Schwartz SB, Windsor EA, Roman AJ, Sumaroka A, Steinberg JD, Jacobson SG *et al*: **ABCA4 disease progression and a proposed strategy for gene therapy**. *Hum Mol Genet* 2009, **18**(5):931-941.

9. Allikmets R, Singh N, Sun H, Shroyer NF, Hutchinson A, Chidambaram A, Gerrard B, Baird L, Stauffer D, Peiffer A *et al*: **A photoreceptor cell-specific ATP-binding transporter gene (ABCR) is mutated in recessive Stargardt macular dystrophy**. *Nat Genet* 1997, **15**(3):236-246.

10. Briggs CE, Rucinski D, Rosenfeld PJ, Hirose T, Berson EL, Dryja TP: **Mutations in ABCR (ABCA4) in patients with Stargardt macular degeneration or cone-rod degeneration**. *Invest Ophthalmol Vis Sci* 2001, **42**(10):2229-2236.

11. Lewis RA, Shroyer NF, Singh N, Allikmets R, Hutchinson A, Li Y, Lupski JR, Leppert M, Dean M: **Genotype/Phenotype analysis of a photoreceptor-specific ATP-binding cassette transporter gene, ABCR, in Stargardt disease**. *Am J Hum Genet* 1999, **64**(2):422-434.

12. Collin RW, Safieh C, Littink KW, Shalev SA, Garzozi HJ, Rizel L, Abbasi AH, Cremers FP, den Hollander AI, Klevering BJ *et al*: **Mutations in C2ORF71 cause autosomal-recessive retinitis pigmentosa**. *Am J Hum Genet* 2010, **86**(5):783-788.

13. Dryja TP, Finn JT, Peng YW, McGee TL, Berson EL, Yau KW: **Mutations in the gene encoding the alpha subunit of the rod cGMP-gated channel in autosomal recessive retinitis pigmentosa**. *Proc Natl Acad Sci U S A* 1995, **92**(22):10177-10181.

14. Lotery AJ, Jacobson SG, Fishman GA, Weleber RG, Fulton AB, Namperumalsamy P, Heon E, Levin AV, Grover S, Rosenow JR *et al*: **Mutations in the CRB1 gene cause Leber congenital amaurosis**. *Arch Ophthalmol* 2001, **119**(3):415-420.

15. den Hollander AI, Heckenlively JR, van den Born LI, de Kok YJ, van der Velde-Visser SD, Kellner U, Jurklies B, van Schooneveld MJ, Blankenagel A, Rohrschneider K *et al*: **Leber congenital amaurosis and retinitis pigmentosa with Coats-like exudative vasculopathy are associated with mutations in the crumbs homologue 1 (CRB1) gene**. *Am J Hum Genet* 2001, **69**(1):198-203.

16. Littink KW, van den Born LI, Koenekoop RK, Collin RW, Zonneveld MN, Blokland EA, Khan H, Theelen T, Hoyng CB, Cremers FP *et al*: **Mutations in the EYS gene account for approximately 5% of autosomal recessive retinitis pigmentosa and cause a fairly homogeneous phenotype**. *Ophthalmology* 2010, **117**(10):2026-2033, 2033 e2021-2027.

17. Miano MG, Testa F, Filippini F, Trujillo M, Conte I, Lanzara C, Millan JM, De Bernardo C, Grammatico B, Mangino M *et al*: **Identification of novel RP2 mutations in a subset of X-linked retinitis pigmentosa families and prediction of new domains**. *Hum Mutat* 2001, **18**(2):109-119.

18. Patil SB, Hurd TW, Ghosh AK, Murga-Zamalloa CA, Khanna H: **Functional analysis of retinitis pigmentosa 2 (RP2) protein reveals variable pathogenic potential of disease-associated missense variants**. *PLoS One* 2011, **6**(6):e21379.

19. Zhang F, Zhang Q, Shen H, Li S, Xiao X: **[Analysis of rhodopsin and peripherin/RDS genes in Chinese patients with retinitis pigmentosa]**. *Yan Ke Xue Bao* 1998, **14**(4):210-214.

20. McLaughlin ME, Ehrhart TL, Berson EL, Dryja TP: **Mutation spectrum of the gene encoding the beta subunit of rod phosphodiesterase among patients with autosomal recessive retinitis pigmentosa**. *Proc Natl Acad Sci U S A* 1995, **92**(8):3249-3253.

21. Zernant J, Kulm M, Dharmaraj S, den Hollander AI, Perrault I, Preising MN, Lorenz B, Kaplan J, Cremers FP, Maumenee I *et al*: **Genotyping microarray (disease chip) for Leber congenital amaurosis: detection of modifier alleles**. *Invest Ophthalmol Vis Sci* 2005, **46**(9):3052-3059.

22. Fernandez-Martinez L, Letteboer S, Mardin CY, Weisschuh N, Gramer E, Weber BH, Rautenstrauss B, Ferreira PA, Kruse FE, Reis A *et al*: **Evidence for RPGRIP1 gene as risk factor for primary open angle glaucoma**. *Eur J Hum Genet* 2011, **19**(4):445-451.

23. Aldahmesh MA, Safieh LA, Alkuraya H, Al-Rajhi A, Shamseldin H, Hashem M, Alzahrani F, Khan AO, Alqahtani F, Rahbeeni Z *et al*: **Molecular characterization of retinitis pigmentosa in Saudi Arabia**. *Mol Vis* 2009, **15**:2464-2469.

24. Birch DG, Peters AY, Locke KL, Spencer R, Megarity CF, Travis GH: **Visual function in patients with cone-rod dystrophy (CRD) associated with mutations in the ABCA4(ABCR) gene**. *Exp Eye Res* 2001, **73**(6):877-886.

25. Audo I, Lancelot ME, Mohand-Said S, Antonio A, Germain A, Sahel JA, Bhattacharya SS, Zeitz C: **Novel C2orf71 mutations account for approximately 1% of cases in a large French arRP cohort**. *Hum Mutat* 2011, **32**(4):E2091-2103.

26. Jacobson SG, Cideciyan AV, Iannaccone A, Weleber RG, Fishman GA, Maguire AM, Affatigato LM, Bennett J, Pierce EA, Danciger M *et al*: **Disease expression of RP1 mutations causing autosomal dominant retinitis pigmentosa**. *Invest Ophthalmol Vis Sci* 2000, **41**(7):1898-1908.

27. Astuto LM, Bork JM, Weston MD, Askew JW, Fields RR, Orten DJ, Ohliger SJ, Riazuddin S, Morell RJ, Khan S *et al*: **CDH23 mutation and phenotype heterogeneity: a profile of 107 diverse families with Usher syndrome and nonsyndromic deafness**. *American journal of human genetics* 2002, **71**(2):262-275.

28. den Hollander AI, ten Brink JB, de Kok YJ, van Soest S, van den Born LI, van Driel MA, van de Pol DJ, Payne AM, Bhattacharya SS, Kellner U *et al*: **Mutations in a human homologue of Drosophila crumbs cause retinitis pigmentosa (RP12)**. *Nat Genet* 1999, **23**(2):217-221.

29. Maw M, Kumaramanickavel G, Kar B, John S, Bridges R, Denton M: **Two Indian siblings with Oguchi disease are homozygous for an arrestin mutation encoding premature termination**. *Human mutation* 1998, **Suppl 1**:S317-319.

30. Swain PK, Chen S, Wang QL, Affatigato LM, Coats CL, Brady KD, Fishman GA, Jacobson SG, Swaroop A, Stone E *et al*: **Mutations in the cone-rod homeobox gene are associated with the cone-rod dystrophy photoreceptor degeneration**. *Neuron* 1997, **19**(6):1329-1336.

31. Hanein S, Perrault I, Gerber S, Tanguy G, Barbet F, Ducroq D, Calvas P, Dollfus H, Hamel C, Lopponen T *et al*: **Leber congenital amaurosis: comprehensive survey of the genetic heterogeneity, refinement of the clinical definition, and genotype-phenotype correlations as a strategy for molecular diagnosis**. *Hum Mutat* 2004, **23**(4):306-317.

32. Dreyer B, Brox V, Tranebjaerg L, Rosenberg T, Sadeghi AM, Moller C, Nilssen O: **Spectrum of USH2A mutations in Scandinavian patients with Usher syndrome type II**. *Hum Mutat* 2008, **29**(3):451.

33. McGee TL, Seyedahmadi BJ, Sweeney MO, Dryja TP, Berson EL: **Novel mutations in the long isoform of the USH2A gene in patients with Usher syndrome type II or non-syndromic retinitis pigmentosa**. *J Med Genet* 2010, **47**(7):499-506.

34. Dharmaraj S, Leroy BP, Sohocki MM, Koenekoop RK, Perrault I, Anwar K, Khaliq S, Devi RS, Birch DG, De Pool E *et al*: **The phenotype of Leber congenital amaurosis in patients with AIPL1 mutations**. *Arch Ophthalmol* 2004, **122**(7):1029-1037.

35. Weston MD, Eudy JD, Fujita S, Yao S, Usami S, Cremers C, Greenberg J, Ramesar R, Martini A, Moller C *et al*: **Genomic structure and identification of novel mutations in usherin, the gene responsible for Usher syndrome type IIa**. *Am J Hum Genet* 2000, **66**(4):1199-1210.

36. Lotery AJ, Namperumalsamy P, Jacobson SG, Weleber RG, Fishman GA, Musarella MA, Hoyt CS, Heon E, Levin A, Jan J *et al*: **Mutation analysis of 3 genes in patients with Leber congenital amaurosis**. *Arch Ophthalmol* 2000, **118**(4):538-543.

37. Sullivan LS, Bowne SJ, Seaman CR, Blanton SH, Lewis RA, Heckenlively JR, Birch DG, Hughbanks-Wheaton D, Daiger SP: **Genomic rearrangements of the PRPF31 gene account for 2.5% of autosomal dominant retinitis pigmentosa**. *Invest Ophthalmol Vis Sci* 2006, **47**(10):4579-4588.

38. Wang L, Ribaudo M, Zhao K, Yu N, Chen Q, Sun Q, Wang Q: **Novel deletion in the pre-mRNA splicing gene PRPF31 causes autosomal dominant retinitis pigmentosa in a large Chinese family**. *Am J Med Genet A* 2003, **121A**(3):235-239.

39. Rosenberg T, Klie F, Garred P, Schwartz M: **N965S is a common ABCA4 variant in Stargardt-related retinopathies in the Danish population**. *Mol Vis* 2007, **13**:1962-1969.

40. Gruning G, Millan JM, Meins M, Beneyto M, Caballero M, Apfelstedt-Sylla E, Bosch R, Zrenner E, Prieto F, Gal A: **Mutations in the human peripherin/RDS gene associated with autosomal dominant retinitis pigmentosa**. *Hum Mutat* 1994, **3**(3):321-323.

41. Dryja TP, Hahn LB, Cowley GS, McGee TL, Berson EL: **Mutation spectrum of the rhodopsin gene among patients with autosomal dominant retinitis pigmentosa**. *Proc Natl Acad Sci U S A* 1991, **88**(20):9370-9374.

42. Bascom RA, Liu L, Heckenlively JR, Stone EM, McInnes RR: **Mutation analysis of the ROM1 gene in retinitis pigmentosa**. *Hum Mol Genet* 1995, **4**(10):1895-1902.

43. Martinez-Mir A, Vilela C, Bayes M, Valverde D, Dain L, Beneyto M, Marco M, Baiget M, Grinberg D, Balcells S *et al*: **Putative association of a mutant ROM1 allele with retinitis pigmentosa**. *Hum Genet* 1997, **99**(6):827-830.

44. Michaelides M, Chen LL, Brantley MA, Jr., Andorf JL, Isaak EM, Jenkins SA, Holder GE, Bird AC, Stone EM, Webster AR: **ABCA4 mutations and discordant ABCA4 alleles in patients and siblings with bull's-eye maculopathy**. *Br J Ophthalmol* 2007, **91**(12):1650-1655.

45. Cremers FP, van de Pol DJ, van Driel M, den Hollander AI, van Haren FJ, Knoers NV, Tijmes N, Bergen AA, Rohrschneider K, Blankenagel A *et al*: **Autosomal recessive retinitis pigmentosa and cone-rod dystrophy caused by splice site mutations in the Stargardt's disease gene ABCR**. *Hum Mol Genet* 1998, **7**(3):355-362.

46. Hernan I, Gamundi MJ, Planas E, Borras E, Maseras M, Carballo M: **Cellular expression and siRNA-mediated interference of rhodopsin cis-acting splicing mutants associated with autosomal dominant retinitis pigmentosa**. *Invest Ophthalmol Vis Sci* 2011, **52**(6):3723-3729.

47. Reig C, Alvarez AI, Tejada I, Molina M, Arostegui E, Martin R, Antich J, Carballo M: **New mutation in the 3'-acceptor splice site of intron 4 in the rhodopsin gene associated with autosomal dominant retinitis pigmentosa in a Basque family**. *Hum Mutat* 1996, **8**(1):93-94.

48. Passerini I, Sodi A, Giambene B, Mariottini A, Menchini U, Torricelli F: **Novel mutations in of the ABCR gene in Italian patients with Stargardt disease**. *Eye (Lond)* 2010, **24**(1):158-164.

49. Sohocki MM, Bowne SJ, Sullivan LS, Blackshaw S, Cepko CL, Payne AM, Bhattacharya SS, Khaliq S, Qasim Mehdi S, Birch DG *et al*: **Mutations in a new photoreceptor-pineal gene on 17p cause Leber congenital amaurosis**. *Nat Genet* 2000, **24**(1):79-83.

50. Pennesi ME, Stover NB, Stone EM, Chiang PW, Weleber RG: **Residual electroretinograms in young Leber congenital amaurosis patients with mutations of AIPL1**. *Invest Ophthalmol Vis Sci* 2011, **52**(11):8166-8173.

51. Perrault I, Delphin N, Hanein S, Gerber S, Dufier JL, Roche O, Defoort-Dhellemmes S, Dollfus H, Fazzi E, Munnich A *et al*: **Spectrum of NPHP6/CEP290 mutations in Leber congenital amaurosis and delineation of the associated phenotype**. *Hum Mutat* 2007, **28**(4):416.

52. Brancati F, Barrano G, Silhavy JL, Marsh SE, Travaglini L, Bielas SL, Amorini M, Zablocka D, Kayserili H, Al-Gazali L *et al*: **CEP290 mutations are frequently identified in the oculo-renal form of Joubert syndrome-related disorders**. *Am J Hum Genet* 2007, **81**(1):104-113.

53. Testa F, Rossi S, Sodi A, Passerini I, Di Iorio V, Della Corte M, Banfi S, Surace EM, Menchini U, Auricchio A *et al*: **Correlation between photoreceptor layer integrity and visual function in patients with Stargardt disease: implications for gene therapy**. *Invest Ophthalmol Vis Sci* 2012, **53**(8):4409-4415.

54. Perrault I, Rozet JM, Gerber S, Ghazi I, Ducroq D, Souied E, Leowski C, Bonnemaison M, Dufier JL, Munnich A *et al*: **Spectrum of retGC1 mutations in Leber's congenital amaurosis**. *Eur J Hum Genet* 2000, **8**(8):578-582.

55. Perrault I, Rozet JM, Calvas P, Gerber S, Camuzat A, Dollfus H, Chatelin S, Souied E, Ghazi I, Leowski C *et al*: **Retinal-specific guanylate cyclase gene mutations in Leber's congenital amaurosis**. *Nat Genet* 1996, **14**(4):461-464.

56. Stone EM: **Leber congenital amaurosis - a model for efficient genetic testing of heterogeneous disorders: LXIV Edward Jackson Memorial Lecture**. *Am J Ophthalmol* 2007, **144**(6):791-811.

57. Perrault I, Hanein S, Gerard X, Delphin N, Fares-Taie L, Gerber S, Pelletier V, Merce E, Dollfus H, Puech B *et al*: **Spectrum of SPATA7 mutations in Leber congenital amaurosis and delineation of the associated phenotype**. *Hum Mutat* 2010, **31**(3):E1241-1250.

58. Morimura H, Fishman GA, Grover SA, Fulton AB, Berson EL, Dryja TP: **Mutations in the RPE65 gene in patients with autosomal recessive retinitis pigmentosa or leber congenital amaurosis**. *Proc Natl Acad Sci U S A* 1998, **95**(6):3088-3093.

59. Dryja TP, Adams SM, Grimsby JL, McGee TL, Hong DH, Li T, Andreasson S, Berson EL: **Null RPGRIP1 alleles in patients with Leber congenital amaurosis**. *Am J Hum Genet* 2001, **68**(5):1295-1298.

60. Li Y, Wang H, Peng J, Gibbs RA, Lewis RA, Lupski JR, Mardon G, Chen R: **Mutation survey of known LCA genes and loci in the Saudi Arabian population**. *Invest Ophthalmol Vis Sci* 2009, **50**(3):1336-1343.

61. Sung CH, Davenport CM, Hennessey JC, Maumenee IH, Jacobson SG, Heckenlively JR, Nowakowski R, Fishman G, Gouras P, Nathans J: **Rhodopsin mutations in autosomal dominant retinitis pigmentosa**. *Proc Natl Acad Sci U S A* 1991, **88**(15):6481-6485.

62. Allikmets R: **Further evidence for an association of ABCR alleles with age-related macular degeneration. The International ABCR Screening Consortium**. *Am J Hum Genet* 2000, **67**(2):487-491.

63. Morimura H, Saindelle-Ribeaudeau F, Berson EL, Dryja TP: **Mutations in RGR, encoding a light-sensitive opsin homologue, in patients with retinitis pigmentosa**. *Nat Genet* 1999, **23**(4):393-394.
