# Supplementary material for: Increasing the Yield in Targeted Next-Generation Sequencing by Implicating CNV Analysis, Non-Coding Exons and the Overall Variant Load: The Example of Retinal Dystrophies
Source: PLoS One. 2013 Nov 12;8(11):e78496. doi: 10.1371/journal.pone.0078496 (PMC3827063; doi:10.1371/journal.pone.0078496)
Supplement: File S1 — contains the following files. Figure S1. CNVs (from partial to complete gene deletions) of PRPF31 detected by analysis of NGS data. A heterozygous deletion of all 14 PRPF31 exons was identified in patient 113. In patient 116, exons 1–5 were deleted on one gene copy (the non-coding exon 1 was not yet included in target enrichment and subsequent NGS, but its deletion was confirmed by MLPA in both patients). The dashed line and red arrows indicate lower coverage for heterozygously deleted regions compared to one control sample. Figure S2. Validation of CNVs predicted from NGS data by MLPA. Only confirmed CNVs were considered true CNVs. A. Heterozygous deletion of exon 1 in the EYS gene in patient 57 and his father. B. Heterozygous deletion of exon 4 in the CRX gene in patient 110 and his father. C. Heterozygous deletion of exons 1–14 in the PRPF31 gene in patient 113 and of exons 1–5 in patient 116. RPA: Relative peak area of the patient result file (green) and of the control result files (blue) with standard deviation (error bar). The ratio RPA was calculated as the RPA of the patient versus controls. Deletions are indicated if the ratio RPA falls below 75%. Table S1. Genes analyzed in this study. A. arRP, adRP and LCA genes that were captured and subjected to NGS in this study. B. Functional categorization of genes with causative mutations. Table S2. Additional variants classified as “likely pathogenic”. Classification as pathogenic by at least three out of five bioinformatic prediction programs and a minor allele frequency below 3% in unresolved patients. Although a contribution of these variants to the phenotype cannot be excluded, they were not considered causative. In many cases, they represented monoallelic variants in recessive genes which would not sufficiently explain the phenotype. References S1. References for Table 1 and Table S2. (ZIP) [file pone.0078496.s001.zip › Additional Data Files/Table S1.doc]

**Additional data file 1**

**(a)** Genes for arRP, adRP and LCA captured and subjected to NGS in this study.

| **Gene** | **adRP** | **arRP** | **LCA** | **Gene accession no.** | **OMIM no.** |
| --- | --- | --- | --- | --- | --- |
| *ABCA4* |  | x |  | NM_000350.2 | 601691, 601718 |
| *AIPL1* |  |  | x | NM_014336.3 | 604392, 204000 |
| *ARL6* |  | x |  | NM_032146.3 | 608845, 613575 |
| *BEST1* | x | x |  | NM_001139443.1, NM_004183.3 | 607854, 613194 |
| *C2ORF71* |  | x |  | NM_001029883.2 | 613425, 613428 |
| *CA4* | x |  |  | NM_000717.3 | 114760, 600852 |
| *CEP290* |  |  | x | NM_025114.3 | 610142, 611755 |
| *CERKL* |  | x |  | NM_001030311.2 | 608381, 608380 |
| *CNGA1* |  | x |  | NM_001142564.1 | 123825, 613756 |
| *CNGB1* |  | x |  | NM_001297.4, NM_001135639.1 | 600724, 613767 |
| *CRB1* |  | x | x | NM_201253.2 | 604210, 600105, 613835 |
| *CRX* | x |  | x | NM_000554.4 | 602225, 613829 |
| *EYS* |  | x |  | NM_001142800.1 | 602772 |
| *FSCN2* | x |  |  | NM_001077182.2 | 607643, 607921 |
| *GUCA1B* | x |  |  | NM_002098.5 | 602275, 613827 |
| *GUCY2D* |  |  | x | NM_000180.3 | 600179 , 204000 |
| *IDH3B* |  | x |  | NM_006899.3, NM_174855.2, NM_001258384.1 | 604526, 612572 |
| *IMPDH1* | x |  | x | NM_000883.3 | 146690, 180105, 613837 |
| *KLHL7* | x |  |  | NM_001031710.2, NM_001172428.1 | 611119, 612943 |
| *LCA5* |  |  | x | NM_181714.3 | 611408, 604537 |
| *LRAT* |  | x | x | NM_004744.3 | 604863, 613341 |
| *MERTK* |  | x | x | NM_006343.2 | 604705, 613862 |
| *NR2E3* | x | x |  | NM_016346.2, NM_014249.2 | 604485, 268100, 611131 |
| *NRL* | x | x |  | NM_006177.3 | 162080, 613750 |
| *PCDH21* |  | x |  | NM_033100.2, NM_001171971.1 | 609502, 613660 |
| *PDE6A* |  | x |  | NM_000440.2 | 180071, 613810 |
| *PDE6B* |  | x |  | NM_000283.3 | 180072, 613801 |
| *PDE6G* |  | x |  | NM_002602.3 | 180073, 613582 |
| *PRCD* |  | x |  | NM_001077620.2 | 610598, 610599 |
| *PROM1* |  | x |  | NM_006017.2 | 604365, 612095 |
| *PRPF3* | x |  |  | NM_004698.2 | 607301, 601414 |
| *PRPF31* | x |  |  | NM_015629.3 | 606419, 600138 |
| *PRPF8* | x |  |  | NM_006445.3 | 607300, 600059 |
| *PRPH2* | x |  |  | NM_000322.4 | 179605, 608133 |
| *RBP3* |  | x |  | NM_002900.2 | 180290 |
| *RD3* |  |  | x | NM_183059.2 | 180040, 610612 |
| *RDH12* | x |  | x | NM_152443.2 | 608830, 612712 |
| *RDH5* |  | x | x | NM_002905.3 | 136880, 601617 |
| *RGR* | x | x |  | NM_002921.3 | 600342, 613769 |
| *RHO* | x | x |  | NM_000539.3 | 180380, 613731, 136880 |
| *RLBP1* |  | x |  | NM_000326.4 | 180090 |
| *ROM1* | x |  |  | NM_000327.3 | 180721 |
| *RP1* | x | x |  | NM_006269.1 | 180100, 603937 |
| *RP2* | X-RP |  |  | NM_006915.2 | 312600 |
| *RP9* | x |  |  | NM_203288.1 | 607331, 180104 |
| *RPE65* | x | x | x | NM_000329.2 | 180069, 204100, 613794 |
| *RPGR* | X-RP |  |  | NM_000328.2 | 312610, 300029 |
| *RPGRIP1* |  |  | x | NM_020366.3 | 605446, 613826 |
| *SAG* |  | x |  | NM_000541.4 | 181031, 613758 |
| *SEMA4A* | x |  |  | NM_022367.3 | 607292, 610282 |
| *SNRNP200* | x |  |  | NM_014014.4 | 601664, 610359 |
| *SPATA7* |  | x | x | NM_018418.4 | 604232, 609868 |
| *TOPORS* | x |  |  | NM_005802.4 | 609507, 609923 |
| *TTC8* |  | x |  | NM_144596.2 | 608132, 613464 |
| *TULP1* |  | x | x | NM_003322.3 | 602280, 600132, 613843 |

**(b)** Functional categorization of genes with causative mutations.

| **Gene** | **Functional category** |
| --- | --- |
| *C2ORF71* | connecting cilium |
| *CEP290* |  |
| *LCA5* |  |
| *RP1* |  |
| *RP2* |  |
| *RPGR* |  |
| *RPGRIP1* |  |
| *TOPORS* |  |
| *TULP1* |  |
| *USH2A* |  |
| *CNGA1* | phototransduction |
| *GUCY2D* |  |
| *PDE6B* |  |
| *RDH12* |  |
| *RHO* |  |
| *SAG* |  |
| *ABCA4* | visual cycle |
| *LRAT* |  |
| *RDH5* |  |
| *RPE65* |  |
| *CRB1* | cell adhesion/structure |
| *EYS* |  |
| *PRPH2* |  |
| *AIPL1* | nuclear transport, protein trafficking, chaperone activity |
| *CRX* | transcription |
| *MERTK* | phagocytosis |
| *PROM1* | cellular structure |
| *PRPF31* | splicing |
